# Retrocopy contributions to the evolution of the human genome

**DOI:** 10.1186/1471-2164-9-466

**Published:** 2008-10-08

**Authors:** Robert Baertsch, Mark Diekhans, W James Kent, David Haussler, Jürgen Brosius

**Affiliations:** 1 Center for Biomolecular Science and Engineering, University of California Santa Cruz, Santa Cruz, California 95064, USA; 2 Institute of Experimental Pathology, ZMBE, University of Münster, Von-Esmarch-Str. 56, D-48149, Münster, Germany

## Abstract

**Background:**

Evolution via point mutations is a relatively slow process and is unlikely to completely explain the differences between primates and other mammals. By contrast, 45% of the human genome is composed of retroposed elements, many of which were inserted in the primate lineage. A subset of retroposed mRNAs (retrocopies) shows strong evidence of expression in primates, often yielding functional retrogenes.

**Results:**

To identify and analyze the relatively recently evolved retrogenes, we carried out BLASTZ alignments of all human mRNAs against the human genome and scored a set of features indicative of retroposition. Of over 12,000 putative retrocopy-derived genes that arose mainly in the primate lineage, 726 with strong evidence of transcript expression were examined in detail. These mRNA retroposition events fall into three categories: I) 34 retrocopies and antisense retrocopies that added potential protein coding space and UTRs to existing genes; II) 682 complete retrocopy duplications inserted into new loci; and III) an unexpected set of 13 retrocopies that contributed out-of-frame, or antisense sequences in combination with other types of transposed elements (SINEs, LINEs, LTRs), even unannotated sequence to form potentially novel genes with no homologs outside primates. In addition to their presence in human, several of the gene candidates also had potentially viable ORFs in chimpanzee, orangutan, and rhesus macaque, underscoring their potential of function.

**Conclusion:**

mRNA-derived retrocopies provide raw material for the evolution of genes in a wide variety of ways, duplicating and amending the protein coding region of existing genes as well as generating the potential for new protein coding space, or non-protein coding RNAs, by unexpected contributions out of frame, in reverse orientation, or from previously non-protein coding sequence.

## Background

While it is said that the human and chimpanzee genomes share anywhere from 95 to 98.5% similarity in their DNA sequences, base exchanges and small indels alone are unlikely to completely explain the differences among diverging primates and between other mammals. Comparative genomics identifies functional elements by searching for conserved DNA across species and is an excellent method for identifying highly conserved biological functions [1-4]. However, sequence conservation is not sufficient to identify *newly *evolved functions. Although point mutations in combination with selection can explain changes in transcriptomes and proteomes, the high number of retroposition events along the primate lineage must also be considered to understand the phenotypic difference between primates and other mammals. Our aim is to understand how one class of these, retroposed mRNA (which we call retrocopies) influenced gene evolution in primates.

Primates experienced a large increase in retroposed insertions, and at least forty-five percent of the human genome is composed of retroposed elements [5]. The majority of random sequences in the human genome are also retroposition-derived, but are too old to be recognized as such [6]. The subclass of retroposed copies of spliced mRNAs are referred to as retrocopies, irrespective of their potential functionality [7]. Retrogenes are then those retrocopies that do not decay in the genome but have been exapted into a variant or novel function. Discernible retrocopies contribute only about 1% of the human genome, yet they have a much more varied protein coding space than other more numerous types of retroposed elements, including the long interspersed elements (LINEs, 21%) and short interspersed elements (SINEs, 13%). The latter lack protein coding regions altogether. Yet, part of Alu and MIR SINEs contributed to newly evolved exons [8-12].

Authors differ widely in their assessment of the number of functional retrogenes [13-18]. Following the first reports of functional retrogenes in the late eighties [19-21], it was thought that these were isolated cases, as mRNA retrocopies lack their own promoter elements. Despite this apparent handicap, research in the last 20 years has shown that gene duplication via retroposition – as opposed to segmental duplication of entire genes including their regulatory elements- is able to equip retrogenes with regulatory elements that are different from those in the parent gene and often leads to different expression patterns [22-24]. Formation of new genes via retroposition followed by positive selection and/or adaptive evolution during modification of genes via exon acquisition has been increasingly reported [7,22,25,26]. In addition to simple duplication events, gene fusions involving retrocopies that contribute protein domains with sequence similarities to existing genes were also reported both in animals [7,27,28] and in plants [26]. Transcribed retrogenes or retrocopies also have the potential to become functional as non-protein coding RNAs (reviewed by Zheng and Gerstein [14] and Sasidharan and Gerstein [29]).

The number of new genes that have arisen since mouse and human split from their common ancestor is small compared with the total number of human genes [30] and apparently, most proteins arose by duplication and divergence of existing ones [31]. Yet, it would be surprising if evolution would not have grasped opportunities of genes (or parts therof) "out of the blue", i.e., from loci that previously were intergenic or intronic. Early studies suggested "overprinting" of a protein coding region in a different reading frame as a means of generating new protein sequence space [32-34]. This concept has been revisited one and a half decades later in alternatively spliced genes [35]. The idea of recruiting novel protein sequence space out of random intronic sequences dates back to Wally Gilbert's suggestion three decades ago [36] with experimental evidence initially accumulating slowly [11,37-39]. Interestingly, it has been shown experimentally that an arbitrary sequence can evolve towards acquiring a biological function [40]. Young genes or young parts of more ancient genes are a unique set to examine because we can see both the putative successes and apparent failures of natural selection, before the latter are erased by mutations. Although our study focuses on these more recent events, we also find evidence for more ancient events. This, along with the fact that 12–15% of mammalian genes are intronless [41], one of the hallmarks of retrocopy retroposition, suggests that the process of retroposition and the idiosyncratic variations of potential novel protein sequence acquisition have been important for billions of years in generating novel protein-sequence space [36,42,43]. Our results document that there are many mechanisms beyond segmental duplication and point mutations by which genomes generate new genes or variants of existing ones. Retrocopies provide the means for modifying splice patterns of genes [44], potentially adding entirely new protein coding sequences, and contributing non-protein coding RNA or regulatory sequences, thereby expanding the possibilities to shape gene evolution. From here on, when we mention retrocopy-derived protein sequences, ORFs, and/or exons, we assert that they are potential, hypothetical or theoretical only. The main rationale of this publication is to delineate the multitude of possible ways in that mRNA retrocopies, once exapted, can contribute to novel protein sequences over evolutionary time.

## Results

### Retrocopies with strong evidence of expression

To determine how many retrocopies are potentially functional, we used BLASTZ to align all human mRNAs to the human genome, which resulted in several hundred thousand alignments. These matches were then scored for a set of features, including the number of processed introns; the absence of conserved splice sites; breaks in orthology with mouse, dog, and rhesus monkey; the presence, position, and length of the poly(A) tail; and sequence similarity and fraction of the parent mRNA that is represented in the retrogene (see Methods for full description), indicating evidence of the likelihood of recent retroposition. From this we obtained a set of 12,801 candidates that are likely retroposed copies of intron-containing parent genes. In order to set our score threshold, we compared our set to the manually curated Vega of processed pseudogenes (retrocopies of mRNAs that may or may not be functional). When we found disagreements between the sets, we either improved our feature set or discovered problems with the Vega annotation. This resulted in improvements of both. In order to determine if the retrocopies are expressed, we looked for overlap with mRNA or EST evidence. Following filtering to eliminate 6413 cases without mRNA or EST evidence, we found that 6,287 retrocopies showed evidence of expression by at least one EST or one mRNA [See Additional File 1]. For our analysis we used more stringent requirements for expression (see below) than previous work. We chose not to use Ka/Ks analysis to look for evidence of natural selection, mostly due to the short length of retrogene sequences. Many of those could be functional parts of genes; however, for more recent events, final proof might be difficult.

### Major categories of retrocopy contributions

To evaluate the types of events that led to new functional gene candidates or modifications of existing genes, and to reduce the possibility that a given transcript resulted from genomic priming, we increased the stringency factor for evidence of expression and examined in more detail a reduced set of 726 cases that overlapped at least five ESTs and one mRNA or annotated as a gene in RefSeq or UCSC KnownGenes (derived from Swiss-Prot).

We examined, in detail, all cases that were not purely duplication events, specifically those events that exhibited evidence of exon acquisition (see Methods): 1) cases with multiple coding exons, and 2) cases that showed evidence of contributions from retrocopy ORFs in the antisense direction. In general, we found that the retroposition events can be described predominantly by the following three categories: Type I: exon acquisition, in which part of the retrocopy was included into an existing gene transcript, in particular, in which a portion of the retrocopy could potentially serve as a protein coding exon. Type II: retropositional gene duplication, in which apparently no pre-existing host gene at the site of insertion was altered. Type II events, in order be functional, would require recruitment of resident regulatory elements at the site of insertion, such as promoters and/or enhancers, and the process may have been accompanied by intron generation, for example, to reduce the size of 5' UTRs [24]. Finally, Type III retrocopy events occur, in contrast to Type I and II events, when the retrocopy contributed a sequence that is largely out-of-frame, derived from a UTR, or in the opposite orientation with respect to the retrocopy's parent. Other flanking DNA sequences, including those derived from other transposed elements (SINEs, LINEs, endogenous retroviruses, and DNA transposons), may also be co-opted into the structure of these *ab initio *gene candidates. Hence, the Type III genes, if functional, have a protein sequence that is mostly novel.

### Comparison with other datasets

We found good agreement between our candidate set of transcribed retrocopies and the major transcribed retrocopy datasets produced by other groups [7,45,46]. Of the 223 transcribed retrocopies reported by Harrison [45,46] we agreed with 189 cases. Randomly selected cases that were missing from our set were cases that relied soley on scarce EST data and fell below our threshold. Of the ten randomly selected cases in our set that were missing from the Harrison set, 30% were present in the Kaessmann set [7]. Of the remaining cases not present in our set or Kaessman's, 20% have weak expression evidence but were nevertheless classified as retrocopies by Harrison [See Additional File 1]. In contrast, we found many examples in the HOPPSIGEN dataset [47] that were not present in our dataset or Kaessman's set. A random sample of ten were all found to be either segmental duplications or inactive LINE elements that we assume were false positives in their data. Kaessmann reported 1,080 expressed retrocopies with at least one EST [7]. We agreed with 936 of these. Most of the cases missing from our set had mitochondrial, immunoglobin or zinc finger genes as parent genes. These were systematically excluded from our dataset because they are frequently generated by a different mechanism, i.e., segmental duplication. We reported 936 cases that were not present in Kaessman's set. Most were due to the smaller starting gene set that his pipeline used and exclusion of parental UTRs from the analysis.

Although the functional potential of Type II retrogenes was discussed early on [23,24] and overwhelmingly substantiated over the past 10 years [7,18,22,48], only a few Type I exon-acquisition events have been reported [49] as well as de-novo gene evolution [50-52]. The significant number of Type I and Type III events that we report demonstrates the extent of the contribution of retrocopies to the evolutionary processes that test, reject, and retain novel amino acid encoding sequence space. All the retrogene candidates fall along a continuum from a large degree of similarity (Type II) to little similarity (Type III) to the original sequences in their respective parent genes. Many of the putative, novel retrogenes, potentially encoding proteins with no similarities to other existing proteins, may have been missed by methods relying on protein alignments, as protein-based screening methods cannot find antisense insertions and also are not able to align UTR regions of retrogenes. Protein alignment methods miss Type III retrogenes entirely. The retrocopies involved in generation of Type III gene candidates made relatively small, but potentially 'seeding', contributions to the formation of novel genes. Of course, it must be emphasized that most of the Type II and Type III mRNA retrocopy-derived "retrogenes" described in this study are putative genes for which no proteins have as yet been documented. While some of these new transcripts may code for proteins, others may serve as non-protein coding RNAs, possibly involved in cellular regulation [14,29] or in chromatin remodeling [53].

### Overview of types of events whose expression was strongly supported

Of the 726 candidate retrocopies whose expression was supported by many transcripts, 624 were composed of single protein coding exons and 102 contained multiple protein coding exons. The 102 cases came from a set of manually examined cases that overlapped known genes with more than one exon (about 500 cases), single exon cases transcribed in the reverse orientation based on EST and mRNA evidence (32 cases), and a random sample of the other single exon cases that slipped through our initial screen due to alternative splicing (see Table 1). We compared: 1) the phylogenetic conservation of the ORF in the various species listed in Methods, 2) the relative contribution of the retrocopy to the new gene, 3) the relative contribution of the host gene (where applicable), 4) contribution from other types of transposed elements, 5) whether the retrocopy inserted in the sense or antisense orientation, and 6) we compared the parent ORF to the retrocopy ORF looking for frameshifts and mutations. Conclusions based on phylogenetic analysis are to be treated with caution as the non-human primate sequences contain a sufficient percentage of mistakes, erroneously indicating lack or presence of phylogenetic conservation in predicted ORFs.

**Table 1:** Description and distribution of expressed retrocopy events

Type of event	Parent gene contribution	Count	Percentage
Type I – Exon acquisition (host gene modified by retrocopy)	New 5' exon (UTR and/or N-terminal protein coding)	10	1%

	New 3' exon (UTR and/or C-terminal protein coding)	18	2%

	New internal exon	6	1%

Type II – duplication (no host gene involved)	Single exon	624	86%

	Exons/introns generated, post insertion	55	8%

Type III – novel genes (no host gene involved)	Antisense, majority of ORF out-of-frame wrt to parent, and other cases (e.g., from non-genic regions)	13	2%

Total		726	

Type I: retrocopy inserted into or near an existing gene. A portion of the retrocopy contributes, mostly by alternative splicing, a new sequence to a pre-existing mRNA. Type I events can be divided into cases that add new N- or C-terminal encoding exons or internal exons. Type II: duplicated gene inserted at a locus where no prior gene existed. Type II events often acquired 5' or 3' UTR portions from the locus of integration after the insertion. Type III: novel gene sequence, whose encoded protein has little or no amino acid sequence similarity to that of the retrocopy's parent. Frequently, Type III events include SINEs, LINEs, LTRs etc., as well as unannotated sequences as additional contributors to gene candidates.

### Type I – genes modified by exon acquisition

**Figure 1:**
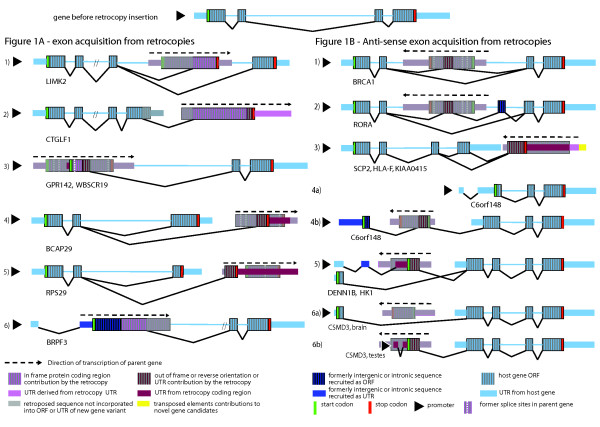
**Categories of Type I retrocopy events**. A. Examples of Type Ia exon acquisitions contributed by "same orientation" of retrocopies (in magenta or dark red) with respect to host gene (light blue); not drawn to scale, splice events are marked by angled lines, open reading frames are depicted as vertically striped thick bars, UTRs by medium size bars, introns in the host gene as light blue lines (for symbols and colors, see also keys below). When parts of retrocopies are described they correspond to what they used to be in the parent gene. The retrocopy's start and stop codons are shown by green and red vertical bars, respectively. Retrogene parts apparently not recruited as functional modules are overlayered with gray. B, Examples of Type Ib exon acquisitions contributed by "reverse orientation" retrocopies. For detailed descriptions, see text.

Of all cases with strong evidence of expression that we inspected, we identified 5% as being potential gene fusions, or exon-acquisition events. It is generally assumed that inserted retrocopies decay without affecting the structure of the host gene. However, we found several examples in which part of a retrocopy ORF integrated into the host gene (Figure 1A, categories 1–4, 6; Table 2), and often led to alternative mRNA splicing (Figure 1A, categories 1, 4, 5). We cannot be sure of the duration of time between the retrotranposition event and the start of alternative splicing. The new splice sites were either fortuitously present in the ORF of the retrocopy, or they arose subsequent to the integration by base changes over time. We did not observe splice sites in retrogenes that coincided with the splice sites in the parent gene. This is not surprising, as important intronic parts of splice sites are removed on the processed mRNA templates prior to retroposition. Therefore, Figure 1 shows generic splice sites as dotted white vertical lines that do not coincide with splice sites used in the novel gene context. The six categories in Figure 1A are defined as follows: 1) Part of protein coding sequence from parent is used as alternatively spliced exon of the host gene. 2) Retrocopy contributes new 3' exon to host gene (mostly in-frame, magenta, and partially out-of-frame, dark red, with respect to parent gene). 3) In-frame contribution (magenta) combined with out-of-frame contribution (dark red) form a new N-terminal encoding region. A short 5' UTR (medium size bar, dark red) has been generated from the ORF of the retrocopy.

**Table 2:** Type I retrocopy-exon acquisition events

**Parent Gene/RetroGene**	**Fig**	**Evolutionary Event and possible consequences**	**Evidence**	**Hs**	**Pan**	**Pon**	**Rhesus**	**Marm**
*PPP1R14BLIMK2*	1A-1	Retro adds PKC-activated phosphatase-1 inhibitor domain to LIM, Zinc binding, PDZ kinase gene at alternatively spliced C-terminal exon.	2 spliced mRNAs, 4 spliced ESTs Reviewed RefSeq	686 aa	y but stop codon after 13 aa	y but stop codon after 19 aa	n	n

*CENTG2/CTGLF1*	1A-2	Retro adds centaurin domain to C-terminal of cyclin gene,.	2 spliced mRNAs, 2 spliced ESTs	663 aa	y but seq. gap	Y	y 3 and 63 bp insertion, but 3 in-frame stops, occurring after 450 aa	n

*C10ORF26/BCAP29*	1A-3	14 aa alternative 3' exon sense orientation out of frame wrt coding region of parent	6 spliced ESTs	244 aa	y 244 aa	y 233 aa	y 241 aa	Not assembled

*RPL32/RPS29*	1A-4	Retro insertion triggered new alt spliced C-terminal exon for RPS29. Most of the retro became 3' UTR.	1 spliced mRNA, > 20 spliced ESTs	67 aa	y 67aa	y 67 aa	y 64aa	y 81 aa

*MLTT6/AF1/BRPF3*	1A-5	Retro contributed PHD/zinc finger with bromodomain.	3 spliced mRNAs, > 10 spliced ESTs	1205 aa	y 1205 aa	y 1205 aa	y 1006 aa	y 885 aa

*ATP5/GPR142*	1A-6	Retro swapped in C-terminal portion of GPCR. New ligand in primates?	2 spliced mRNAs, 2 spliced ESTs	462 aa	y but frameshift early in ORF	y but frameshift early in ORF	y 462 aa	y but frameshift early in ORF

*RPL21/BRCA1*	1B-1	Antisense internal cassette (alt spliced) exon inserted by retro.	1 spliced mRNA	1354 aa	y 100% open	y 100% open	y 100% open	n

*CYCS/RORA*	1B-2	Antisense alt. spliced internal cassette exon contributed by retro. Protein evidence in Swiss-Prot and PDB	1 spliced mRNA PDB 1N83,1SOX	556 aa	y in frame stop in first 20 aa	y 556 aa	y late translational start (31aa shorter)	y difficult to check ORF.

*RRAS2/SCP2*	1B-3	Antisense retro triggered shorter alternative transcript in apes.	2 spliced mRNAs, 3 spliced ESTs	332 aa	y 332aa	N	n	n

*FLJ10324/KIAA0415*	1B-3	Antisense, ancient	8 spliced mRNAs	807 aa	y	Seq gap	y	

/*HLA-F*	1B-3	Antisense, alt. spliced C-terminus	2 spliced mRNAs	377 aa	y		y	

*EIF3S6/C6orf148*	1B-4	Retro contributed internal 15 aa antisense exon.	2 spliced mRNAs, 5 spliced ESTs	238 aa	y 100% open		y 100% open	y no ORF splice site has indel

*RPS15A/HK1*	1B-5	Antisense retro triggered slightly later start via novel exon. Alternative translation initiation. Reviewed RefSeq.	1 spliced mRNA 2 spliced ESTs	905 aa	y 905 aa	y 100% open	y 905 aa	n/a

*RPL24/DENN1B*	1B-5	Primate specific antisense internal cassette exon generated short alt spliced transcript encoding 396 aa. Possible alternative translation initiation.	6 spliced mRNAs	396 aa	y 396 aa	y ORF ok but missing splice site	y no start codon	

*RPL18/CSMD3*	1B-6	Antisense retro triggered different start via novel exon.	1 spliced mRNA	3667 aa	seq gap	f/s wrt human.	y f/s wrt to human. Same as PPY	

**Figure 2:**
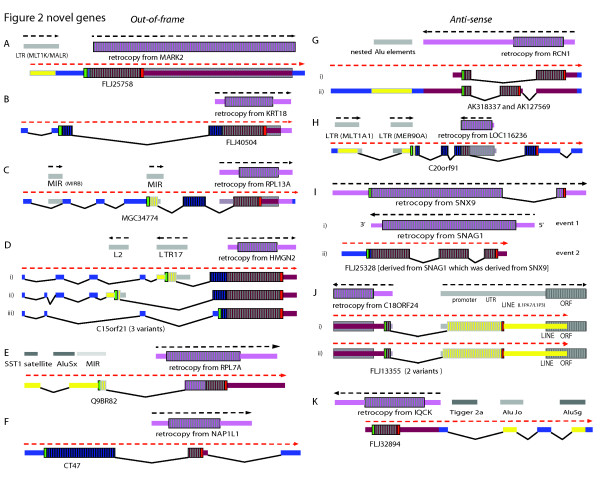
**Novel protein-sequence space generated by parts of retrocopies combined with other transposons or unusual events**. For each part of the figure, the spliced parent mRNA is shown first (before retroposition) and the resulting gene(s) are shown below. New sequence space was triggered by a combination of retrogene insertions, recruitment of non-genic regions including retroposons, whereby the contribution of the retrocopy's original in-frame ORF is very small (see text and legend to Fig. 1 including color key for further details). Yellow boxes with grey vertical stripes and yellow medium size bars correspond to retroposed element contributions to ORFs and UTRs, respectively. For detailed descriptions see text.

Examples of specific categories of retrocopy insertion events shown in Figure 2 with designation of parent gene. Genomic locations of the examples are cross-referenced in [see Additional File 1]. y; indicates presence of the retrogene in this species, n; indicates its absence, aa; amino acids in potential encoded entire protein, f/s; frame shift, wrt; with respect.

We also found several examples in which a putative novel exon had been exapted from an ORF, but the reading frame is now different from that of the parent gene (Figure 1A, category 4) which results in a shorter transcript. Also, putative novel exons were exapted entirely from sequences that correspond to UTRs of the parent gene, here alternatively sliced (Figure 1A, category 5). In other instances insertion of a retrocopy and exaptation of an exon from that sequence triggered recruitment of an additional exon entirely from intronic space. For example, in Figure 1A, category 6, the retrocopy contributes in-frame protein coding region (magenta) combined with unannotated intergenic sequence (dark blue) to form a new N-terminal encoding exon for the host gene. In turn, portions of the intronless retrocopy's protein coding region became an intronic sequence in the host gene (overlayed in grey). An interesting example of retrocopy mediated domain shuffling is the CTGLF1 gene (Figure 1A category 2), which started as a cyclin gene, and then had three domains (PH, ArfGap and Ankyrin) contributed by insertion of a CENTG2-derived retrocopy. The mouse version of this gene, AK132782, has only a cyclin domain and represents the ancestral form before the retrocopy insertion. These observations underscore the fact that natural selection exapts novel sequence space in addition to slowly modifying existing sequence space.

### Type I exon acquisition in reverse orientation

While at least one group has reported on the existence of sense retrocopy integrations into existing genes, with coding contributions [7], this is the first report of mRNA retrocopy integrations in the antisense orientation. The existence of this category of retrocopy events, if functional, supports the idea that natural selection has no preference with respect to the origin of novel sequences. In this category, novel exons were recruited from retrocopies that inserted into or adjacent to host genes in the opposite orientation to the retrocopy's parent gene. As in the case of Type Ia 'sense' retrocopies, the splice sites in the 'antisense' retrocopies, of course, do not correspond to those present in the parent genes. Of the Type Ib examples that we manually inspected, polypyrimidine tracts inserted by the retrocopy – used for recognition of splice sites – were frequently derived from antisense oligopurine tracts in the parent gene. These sequences are often rich in codons for lysine, glutamic acid, and glycine, as well as certain codons of arginine in the parent gene. A few Type Ib examples are described in detail below.

1) Internal exon (dark red) added to host gene in the opposite orientation relative to the parent of the retrocopy For example, the BRCA1 gene has an alternatively spliced internal cassette exon (potentially encoding 22 aa) contributed by RPL21 in the antisense direction (Figure 1B, example 1; Table 2). The insertion occurred after the New World monkey split and the reading frame is open in chimpanzee, orangutan, and rhesus monkey.

2) Internal exon added to host gene triggered recruitment of an additional protein coding exon from formerly intronic sequence (dark blue). For example, RORA acquired an internal cassette (encoding 25 aa – PDB structures 1N83 and 1S0X and Swiss-Prot P35398) from an antisense retrocopy of CYCS. Interestingly, a second exon (encoding 27 aa) appeared in conjunction with the retrocopy-derived exon, apparently derived from an intronic sequence, that maintains the frame of the gene (Figure 1B, example 2). The open reading frame is maintained in orangutan, rhesus monkey, and marmoset, but there is an early in-frame stop in chimpanzee (confirmed by our re-sequencing, unpublished) – an example in which one lineage did not retain such an innovation (see discussion).

3) Recruitment of a 3' exon including novel ORF and 3' UTR generated from ORF of the retrocopy; it extends the ORF of the host gene (Figure 1B, example 3). Examples are SCP2, HLA-F, and KIAA0415 with potentially functional variants that have alternatively spliced 3' ends that are derived from antisense retrocopy insertions of RRAS2, RPL23, and FLJ10324, respectively. The SCP2 variant that includes the retrocopy-derived exon has a shorter transcript (potentially encoding 338 aa instead of 547 aa) and is only present in chimpanzee and human. In HLA-F the insertion generated a longer ORF (potentially encoding 442 aa instead of 362 aa; Figure 1B, example 3). Importantly, the retro-derived variants of SCP2 and HLA-F are reviewed NCBI Refseq genes.

4) Portion of the retrocopy contributes a potentially alternatively spliced protein coding exon in conjunction with a novel protein coding exon generated from intergenic sequence (dark blue). For C6orf148, we detected two mRNA variants; the first, depicted in Figure 1B (example 4a), presumably also represents the ancestral status prior to the retrocopy insertion. The second putative variant has an alternative, upstream promoter, a new first exon from an unknown source, and a second protein coding exon (Figure 1B, example 4b) derived from the EIF3S6 retrocopy in reverse orientation. Surprisingly, the third putative coding exon in the second variant is also longer than the corresponding N-terminal coding region in the original variant. Part of the EIF3S6 UTR was potentially exapted as a protein coding sequence (example 4a). Both splice forms and open reading frames coexist in chimpanzee and rhesus monkey.

5) Alternative splicing or alternative translation after retrocopy insertion. The first putative protein coding exon becomes one of the 5' UTR exons (light blue); a second 5' UTR exon is recruited from unknown sequences. The first putative protein coding exon (dark red) is recruited from the retrocopy (Figure 1B, example 5). As in the aforementioned examples, DENN1B and HK1 also exhibit mRNA variants with and without their respective retrocopies. Their promoters are shared by both variants and both have putative alternative translation starts. Interestingly, in both cases the version with retrocopy contribution does not start transcription in the first exon, but instead, includes a second UTR exon before splicing to the retrocopy-derived putative antisense coding exon. The next protein coding exon is shared by both variants. The ORF for HK1 is open in chimpanzee, orangutan, and rhesus monkey. DENN1B has valid ORFs only in human and chimpanzee, but the retrocopy-derived portion is present with disruptions in orangutan and rhesus monkey.

6) Two 5' UTR exons, intron, and N-terminal encoding exon are recruited from the protein coding region of the retrocopy. For example, one variant of CSMD3 is expressed in the brain and contains a sequence encoding a potential N-terminal 79 aa exon (Figure 1B, example 6a). The other putative variant of CSMD3 is expressed in testes (based on mRNA and EST evidence), and instead of the 79 aa exon uses part of an antisense RPL18-derived retrocopy; the event might also have led to use of a new promoter for the gene (Figure 1B, example 6b). In addition, a 5' UTR exon and a small intron were derived from the protein coding region of the retrocopy. Only the human version of the retrocopy-containing putative variant has an intact ORF.

### Type II duplication events

Of the Type II events, 60 of them contained one or more 5' and/or 3' untranslated exons acquired along with regulatory elements derived from the flanking region of the insertion site [see Additional File 2, categories 2 and 3] as predicted previously [24]. A few examples in which introns also arose in flanking UTRs were reported recently [7]. In our numerous examples, we found no indication that the UTR introns came from the parent gene. Occasionally, a 5' or 3' exon recruited from the locus provided not only a UTR, but also the first or last protein coding exon [see Additional File 3]. We also observed shorter and longer N- or C-terminal encoding parts of genes in separate lineages [see Additional File 2, categories 4–8,10,11], representing one of the mechanisms that might explain the frayed ends of many protein homologs, even orthologs [54]. In addition, we found cases where an intron arose within the coding region after retroposition [see Additional File 2, category 8–11, Additional File 3]. These and the aforementioned examples (categories 2 and 3) underscore the notion that even intron-containing genes, especially those with large exons and relatively intron-impoverished with respect to their parent genes, can be derived from retroposition. Similarly, one or several introns of a gene can be lost by recombination with the corresponding retrocopies [55].

### Type III novel gene candidates

In a small fraction of the cases (16) we examined, a putative new gene with no known homologs included a retrocopy (usually only part thereof) that inserted into the genome and possibly provided protein coding sequence either 1) out-of-frame (Figure 2A–F, Additional File 4A) or 2) antisense with respect to the parent gene (Figure 2G–K, Additional File 4B–E, Table 3 and Additional File 3).

**Table 3:** Type III novel retrogenes that are out of frame or reverse sense with respect to the parent gene

**Parent Gene/Retro**	**Fig**	**Evolutionary Event**	**Evidence of Expression**	**Human**	**Chimp**	**Orangutan**	**Rhesus**	**Marmoset**
*MARK2/FLJ25758*	2A	Out of frame	4 mRNAs and 4 ESTs	81 aa	81 aa	81 aa	96 aa	n

*KRT18/FLJ40504*	2B	Human specific, 2 exons: first out of the blue and second from antisense retro	3 spliced mRNAs, 10 spliced ESTs	259 aa	stop after 114 aa	truncated to 152 aa by f/s, frame shift in ATG disrupts ORF	first exon in sequencing gap f/s 2 stops	n

*RPL13A/NM_203308*	2C	3 coding exons: first from MIR, second unknown source, third from antisense retrocopy	1 spliced mRNAs, 5 spliced ESTs	170 aa	170 aa	y 134 aa early stop, exon1,2 open	partially deleted in rhesus	stop in first 10% of ORF

*HMG2/c15ORF21*	2D	C-terminal exon from retro (partially out of frame), first coding exon from LTR	2 spliced mRNAs, 2 spliced ESTs	172 aa	Disrupted	Disrupted	4 stop codons, 1st at 10 aa.	y 72 aa due to f/s.

*HMG2/c15ORF21*	2D	C-terminal coding exonfrom retro (partially out-of-frame), first coding exon from L2 LINE	4 spliced mRNAs, 17 spliced ESTs	163 aa	Disrupted	Disrupted	4 stop codons, 1st at 10 aa.	y 63 aa due to f/s.

*HMG2/c15ORF21*	2D	Out-of-frame sense retrocopy	4 spliced mRNAs, 17 spliced ESTs	150 aa	Disrupted	Disrupted	4 stop codons, 1st at 10 aa.	y 56 aa due to f/s.

*RPL7A/Q9BR82*	2E	Novel primate specific gene candidate, peri centromeric	1 spliced mRNA, 20 spliced ESTs	89 aa	y 86 aa	Check transMap	y but part of gene inverted	Not assembled

*NAP1L1/CT47*	2F	Primate specific cancer testis gene candidate. Swiss-Prot Q5JQC4	1 spliced mRNA, 10 spliced ESTs	288 aa	seq gap in chimp	291 aa about 80% common with human (ensembl)	y good ORF 15 bp multiple of 3 indel.	287 aa

*RCN1/AF318337*	2G	Antisense retrocopy combined with Alu	1 spliced mRNA, 3 spliced ESTs	123 aa	y 127 aa	n AluY and retro are missing	n	n

*RCN1/AK127569*	2G	Antisense retrocopy combined with Alu	2 spliced mRNAs, > 20 spliced ESTs	61 aa	y 60 aa	n AluY and retro are missing	n	n

*LOC116236 c20ORF91*	2H	Novel gene candidate with 4 coding exons: first from LTR, second and forth unknown sources and third from antisense retrocopy	2 spliced mRNAs, 3 spliced ESTs	154 aa	chimp not assembled in this region	y retro exon is open (assembly not complete)	n	n

*SNAG1/FLJ25328*	2I	Human specific	5 spliced mRNAs, 13 spliced ESTs	205 aa	y 5 stops	y good ORF but gap (or deletion) in first 50aa	y 107 aa, starts later in first exon	y multi exon gene unknown ORF

*c18ORF24/FLJ13355*	2J	Novel 2 exon gene mostly from LINE and antisense retrocopy	4 spliced mRNAs, 6 spliced ESTs	154 aa	y but stop codon after 30 aa	y 137 aa includes LINE	y but short 78 aa	LINE not present

*c18ORF24/FLJ13355*	2J	Novel 2 exon gene mostly from LINE and antisense retrocopy	1 spliced mRNA, 4 spliced ESTs	147 aa	y but stop codon after 30 aa	y 127 aa includes LINE	y but short 78 aa	LINE not present

*IQCK/FLJ32894*	2K	Antisense novel gene, possible NMD	1 spliced mRNA, 2 unspliced mRNA, 4 ESTs	166 aa	y 169 aa	y 169 aa	early in frame stop	n

Details of candidate genes showing parent gene, expression evidence and phylogeny shown in Figure 2. y; indicates presence of the retrogene in this species, n; indicates its absence, aa; amino acids in potential encoded protein, f/s; frame shift, wrt; with respect to.

Examples are briefly described as follows: A) The novel candidate gene FLJ25758 was generated from MARK2-derived retrogene featuring protein coding sequence completely out-of-frame with respect to the parent. There is a potential LTR contribution of promoter and the remaining UTR sequences of FLJ25758 gene were derived from flanking sequence shown as blue bars. B) The novel candidate gene FLJ40504 was generated from a KRT18-derived retrocopy out-of-frame with respect to the parent, the remainder of the protein coding sequence was derived from flanking region. C) The first protein coding exon of candidate gene MGC34774 was derived from a MIR element (yellow); the second exon from intergenic region (blue) and the final protein coding exon from the 5' UTR and ORF of L13A-derived retrocopy out-of-frame (dark red). D) The novel gene candidate C15orf21 is processed into three alternatively spliced transcripts, two of which use retroposed sequences as first protein coding exon (yellow). The second protein coding exon, in part from unknown sequences, was fused to an HMG14-derived retrocopy completely out-of-frame. E) The RPL7A-derived retrocopy contributes half of the ORF in-frame (magenta) and half out-of-frame (dark red) yielding novel gene candidate Q9BR82. Upstream exons were contributed by repeats (yellow). F) CT47 gene was generated, in part, from a NAPL1-derived retrocopy, which contributed the C-terminal encoding region out-of-frame. Most of the ORF (blue) arose from intergenic sequence. G) Two alternatively spliced mRNAs originated from an RCN1-derived retrocopy (3' UTR and ORF) in reverse orientation. H) LTRs contributed UTR and first protein coding exons (yellow); other exons were derived from intergenic sequence (blue); candidate gene C20orf91 contains part of a LOC16236-derived retrocopy in opposite orientation. I) Two events, retroposition of SNX9 followed by a second retroposition of SNAG1 or segmental duplication formed the intron containing candidate gene FLJ25328 in reverse orientation to SNAG1, presumably with generation of two introns out of ancestral ORF sequences. J) A novel gene candidate FLJ13355 (2 splice variants) was formed from a C18orf24-derived retrocopy (retrocopy ORF contributed 5' UTR and retrocopy 5' UTR contributed N-terminal encoding region). The second, larger exon including a large part of ORF was contributed by the internal promoter and 5' UTR region of a LINE element (yellow). K) An IQCK-derived retrocopy contributed the protein coding exon in reverse orientation (dark red) yielding novel gene candidate FLJ32895. Downstream UTR exons were derived from Alu elements and intergenic sequences.

One can summarize the following: Although there is no evidence that a protein is produced, the size of the putative new ORFs ranged from 81 to 259 aa in human, and seven maintained open reading frames in human, chimpanzee and orangutan. Only four cases also had open reading frames in rhesus monkey and only two also in marmoset (Table 3). Suprisingly, all but three had multiple putative exons, lending further weight to the notion that we were not observing random transcription. While fusions between existing genes and mobile elements have been described [56], we also observed exons that were generated, in conjunction with the retrocopy, by other types of transposed elements and/or unannotated sequences. For example, most of the CT47 gene [57] which has evidence of protein coding sequence (Swiss-Prot Q5JQC4) arose initially from unannotated sequence (see below), amplified by segmental duplication and has a valid ORF in human, chimpanzee, orangutan, rhesus macaque, and marmoset. Other cases in which a gene arose from unannotated sequences have been described in flies [50-52]. Out of the 16 primate cases, seven had putative protein coding regions that included repetitive elements [shown in yellow tall boxes, Additional File 4A]. One was composed of a chimeric fusion of two retrocopies [see Additional File 4A, Additional File 3].

### Functional categories of source genes

We looked at GO annotations of the parent genes that spawned retrocopies. For both the expressed on non-expressed sets of retrocopies we found no statistically significant enrichment from a normally distributed set.

## Discussion

The more than 700 instances of evidently transcribed retrocopies in the human genome indicate that this process has contributed significantly to our transcriptome, and may have contributed novel protein coding segments more often than previously appreciated. How these changes altered the content and regulation of the repertoire of protein coding genes remains to be experimentally investigated. However, it is evident that the retrocopy mechanism provides an extremely versatile means to tinker with gene structure, and many of the topologically possible kinds of novel exon recruitment events seem to have been explored.

We observed retroposition events that happened prior to vertebrate diversification, those that date from after the *Homo sapiens *lineage diverged from that of the chimpanzees, and those that occurred at times between these events. Each of these events was/is initially a chance event – probably neutral at best. The combination of neutral forces and natural selection are responsible of the future trajectories, whether such a gene with retrocopy contribution persists or is abandoned in all or some of the subsequent lineages after a "trial period", which can be very short or last for tens of millions of years. This does not differ from genes that arose entirely by segmental duplication. In either case, a certain proportion is eventually discarded in all or some lineages, after being active for some time, while others persist.

Does segmental duplication or retroposition lead to more functional duplicated genes? The fact that some retrocopies lack their own promoters might be construed as a disadvantage. However, the high percentage (as much as 70%) of the genome being transcribed [58] should help neutralize this apparent disadvantage. Moreover, recruitment of a novel promoter might be an advantage over that of an amplified gene that was generated via segmental duplication. In fact, many retrogenes exhibit expression patterns that are drastically different from those of the parent genes [23]. Perhaps the increase in retroposition in primates has allowed greater regulatory flexibility to evolve in a relatively short time.

The uncertainty of a long life after the birth of a gene is not much different from the exonizations of novel sequence domains in "established" host genes. Krull [10] examined the history of five Alu element exonizations by phylogenetic analyses and found that many of these events did not persist in all lineages in which they were exonized. In contrast, of five MIR element exonizations analyzed, four are present and expressed as mRNA in all mammals examined [11,12], and there are likely many more – thus far unproven – MIR exonizations. When comparing the older MIR exonizations with the younger Alu exonizations, it is apparent that over the past billion years, most exonization were transient and, due to low levels or lack of evolutionary pressure, did not persist. As an aside, it appears that exonizations can occur at any time following the SINE insertion. There is one apparently old and previously neutrally evolving MIR that only "recently" was exapted as a protein coding exon in the alpha1 nicotinic cholinergic receptor gene in great apes [11]. Likewise, it is conceivable that a retrocopy or part thereof can be exapted at any stage of decay (see C20orf91 example). At the same time, it is clear that some of the aforementioned Alu exonizations have persisted in some lineages and not in others. The inability to predict whether an event will persist is equally impossible for younger Type II retrocopy insertion events.

Novel exon recruitment often occurs at the ends of genes. Eleven of the 36 Type I (exon acquisition) mRNA retrocopy events added exons at the 3' or 5' ends of the transcripts, while only four transcripts contained coding exons inserted internally, the remaining cases have new UTR exons added to existing genes. This makes sense if one considers that only one splice site needs to be added to extend the ORF at the end of the gene. We also observed that Type II retrogenes, presumably during phases of no or little purifying selection and/or during periods of positive selection, frequently changed in different lineages, mostly with respect to the N- and C-terminal encoding parts of the ORFs (i.e., frayed ends). For example, in one lineage the C-terminal encoding part of the ORF is truncated compared to the orthologous region in another lineage. It might be reasoned that changes at protein extremities are better tolerated than elsewhere in the protein. This is supported by large-scale sequence comparisons of orthologous proteins, in which the terminals vary more than do the rest of the proteins [54,59].

Apart from the acquisition of existing protein domains (Type I events), the acquisition of novel protein sequence space via retroposition (Type III events) plays a role in ab initio formation of genes. Apart from hitherto neutrally evolving sequences (see below under iv), these putatitvely novel genes can include i) ORF exons from what corresponds to 5' and 3' UTRs of the parent genes; ii) ORF exons out-of-frame with respect to the parent gene; iii) ORF exons from any retrocopy part, inserted in the antisense orientation; and iv) ORF exons from intronic sequences or intergenic regions adjacent and in addition to the co-opted retrocopy parts. Extreme cases are those examples in Figure 2, in which the retrocopy did not contribute much novel or pre-existing sequence space, but contributed to the formation of potential genes out of unannotated intergenic regions or retroposons.

## Conclusion

Examining the births of these, as yet, putative retrogenes provides us with important ideas concerning the evolution of older, known genes – or parts therof. Of course, for young events the "gene" status is hard to prove short of experimental verification of a functional protein product, and for older events the history of the gene modules is more difficult to discern. These points notwithstanding, whether or not the examples presented will stand the test of time – either on an evolutionary scale or in the lab (if experiments can be devised that are conclusive), this is clearly another way that novel genes or gene variants can arise. We see evidence of the diversity of tinkering that occurs with genes beyond simple point mutations. The generation of new transcripts with protein coding potential derived from anti-sense retrocopies would be an unexpected contribution to protein evolution, if function can be shown. Thus, retroposition is certainly a mechanism that can help explain the differences that we see in phenotypes between species.

## Methods

### Algorithm for finding candidate retrocopies in the human genome

**Figure 3:**
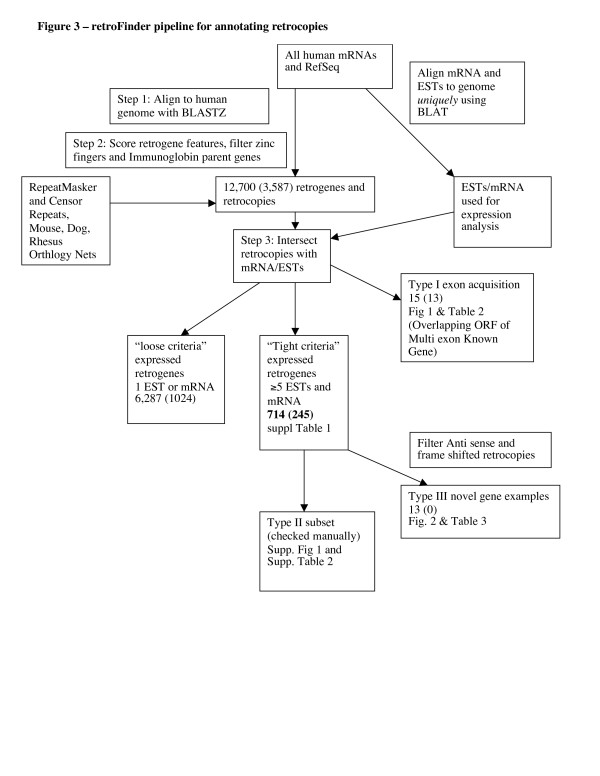
**The retroFinder pipeline for annotating retrocopies**. Alignments of all human mRNAs that aligned more than once to the genome were scored for a set of features (see Methods). Number of strict ESTs, mRNAs, and size of ORF were applied to determine evidence of expression. Retrocopies that partially overlapped the protein coding region of annotated multi exon Refseq genes were classified as exon acquisition events. Numbers in parenthesis were reported previously [7] (Additional files 4, 5, 6, 7).

The search for and identification of retrocopies and their corresponding parent genes have been confounded by the existence of gene duplications generated by other evolutionary processes, such as segmental duplication. To avoid such difficulties we first aligned all human mRNAs (with poly-A tails removed) to the human genome using BLASTZ, and looked for sites where mRNAs aligned in more than one location [60], indicating that one or more gene copies have been made. If one of the locations was annotated as a known gene (referred to as the "parent gene"), we then assigned a confidence score, based on the analysis of a feature vector, to each of the other alignment hits to determine if a retroposition event had occurred. For each putative retrocopy locus, we constructed a feature vector from a number of features (listed below) and used a score function to assign a weight to each feature associated with a retroposition event. A schematic of the entire 'pipeline' is presented in Figure 3.

### Score Function

To exclude segmental duplications (without retrocopy origin) and other non-retroposition related events, we used a score function based on a weighted linear combination of each feature (described below) to evaluate whether a retrocopy event had occurred. For each alignment above, we generated a set of features and applied the score function [see Additional File 3] with a threshold. We trained the weights and the threshold on a set of curated processed pseudogenes (non-functional retrocopies) from the Vega dataset [61]. Vega was chosen since they have a set of carefully manually curated set of pseudogenes using standards used by Sanger in the Vega pseudogene annotation. We used a set of 2,838 processed Vega pseudogenes as positive examples and 303 Vega non-processed psuedogenes as negative examples to set the weights. Chr18 was held out from the Vega training set so that we could check the weights against a set that was not used for training. We then checked high scoring false positives against the Vega set of processed pseudogenes. In some cases we found misannotations in their dataset and they updated their procedure for curation. They discovered that there were differences in how the curators defined pseudogenes that were rectified in subsequent Vega releases (J. Harrow, personal communication).

The following score function was used to combine the features defined below. We used a threshold of 650 to filter the set of 12,801 retrogenes. The threshold was set based on with the Vega processed pseudogene set.

$$retrocopyScore=\sum_{i=0..7}w_{i}f_{i}(x_{i})$$

where functions normalized to scale from 0 to 1000 and weights are between -1 and 1,

f_0_(x) = percent identity to parent gene, w_0 _= +0.3,

f_1_(x) = log_2_(exons removed+1)*200, w_1 _= +0.85,

f_2_(x) = log_2_(chained alignment score)*170 -1000, w_2 _= +0.7,

f_3_(x) = log_2_(length poly A tail +2)*200, w_3 _= +0.4,

f_4_(x) = max(percent coverage of ortholog in mouse/dog) * 10, w_4 _= +0.3,

f_5_(x) = sqrt(count of introns)*750, w_5 _= -1,

f_6_(x) = percent coverage of parent*(1-percent truncated 3') *300, w_7 _= 1,

f_7_(x) = percent overlap repeatMasker (SINES or LINES) *10, w_8 _= -1,

### Description of Features

For each putative retrocopy alignment we extracted the following set of features:

• The most obvious sign of retroposition is the presence of multiple contiguous exons with introns removed. This signal can be weakened by any insertions, deletions, and substitutions that occur after retroposition. We counted the number of contiguous processed exons in the retrocopy and compared that to the parent gene. We did not count any recent Alu/LINE insertions as introns, as that has been a problem with other methods (Zhang, D; personal communication). When we aligned the mRNA to both parent gene and putative retrocopy loci, we were able to map the location of the breaks in the alignment back to the mRNA coordinates. For the parent gene, most of these insertions (larger than 35 bp) corresponded to introns. We made the assumption that if the insertion was larger than 35 bp and it occurred within 7 bp of the splice site in the parent gene, then it was a spliced intron at the retrocopy location. If no splice site was found at this location in the retrocopy and the mRNA was alignable at this location, then it was counted as a "spliced exonn. This feature had the heaviest weight assigned as it is the strongest signal of retrotransposition [see Additional File 3].

• Conserved splice sites were counted by looking at the position of the splice sites in the parent gene in cDNA coordinates. These positions were mapped to the retrogene and any break in the alignment that was larger than 35 bp and within 15 bp of the splice site in the parent gene were considered a retained splice site and reduced the count of the spliced exons. Most retrocopies should not have any conserved splice sites, but occasionally they have retained an intron due to incomplete processing prior to insertion. Therefore this feature's weight was lowered to allow a small number of conserved splice sites provided other signals were strong.

• We counted the number of introns in the retrocopy by looking at gaps in the alignment of the parent cDNA to the retrocopy locus that were larger than 35 bp. If there was no corresponding gap on the cDNA side of the alignment, or if the break in the alignment on the genome side was at least three times larger than the break in the alignment on the cDNA side, then it was counted as an intron and assigned a large negative weight. We masked out Alu/LINE repeat insertions before calculating this feature to avoid counting recent transposons as introns.

• The ''relative orthology with mouse'' feature took advantage of the fact that retrocopies inserted since the mouse/human divergence show a break in the human-mouse genomic alignment as defined by the UCSC syntenic alignment nets [62]. Relative orthology is defined as the ratio of the size of the putative retrocopy to the size of the genomic insertion – defined by the break in the alignment ''net'' (from by the UCSC Browser). Ratios close to one represent possible retroposition events and ratios close to zero are most likely segmental duplications. Non-processed pseudogenes tend to score low because they are often generated via large segmental duplication events. We used the same relative orthology feature with the dog/human and rhesus monkey/human alignment nets to avoid false assignments due to deletions in mouse. The weight on this feature was less than the previous feature since older events will not show a break in orthology.

• The poly(A) tail feature measured the length of the poly(A) tail that was inserted into the genome during retroposition [63]. For more recent insertions, this signal distinguishes retrocopies from non-processed pseudogenes. The poly(A) tail was determined to be the largest scoring segment in a window of 70 bases near the end of the retrocopy. A's were scored +1 and the remaining three bases were scored -1. The weight was rather low, because poly(A) tails can also arise by chance or secondary retroposon insertions, thus, they did not add much weight to the score unless they were quite long.

Since we used a score function to classify retrocopies, the absence of one or two features did not exclude a given candidate. In this way we identified retrocopies that did not show orthology breaks in mammals, which also enabled us to identify older events. Likewise, absence of a poly(A) tail (e.g., in older or truncated retrocopies) did not lead to exclusion. To evaluate the transcriptional competence of the retrocopy set, in addition to the normal criteria used in the UCSC Genome Browser [64-66], we only used cDNA (mRNA) and EST evidence that uniquely mapped to the retrocopy and not to the parent gene in at least five nucleotide positions.

### Filtering Alignments

We removed any candidate retrocopies that overlapped by more than 50% with repeats identified by RepeatMasker [67] and Tandem Repeat Finder [68]. Because RepeatMasker appeared to be overly aggressive in misclassifying pseudogene insertions as repeats, we corrected this by performing a base-by-base intersection with CENSOR [69,70] and eliminated only masking regions where RepeatMasker and CENSOR agreed. We also removed low percentage identity alignments (below 75%) that overlapped Alu elements. We found that recent independent Alus that were close to the parent and the retroposed segment were included in the alignments to the parent genes, generating false alignments, which we discarded manually.

### Determining the Parent Loci

To find the locus of the originating parent gene, we looked for places where the parent mRNA aligned at least twice in the genome and defined the parent location as the best genome hit defined by the UCSC Genome Browser mRNA and refSeq tracks. The other non-overlapping hits define the locations of the retrocopies.

### Resolving Conflicts from Multiple Parent Genes

To resolve conflicts posed by multiple potential parent genes, we initially took the simple approach of labeling as the most likely true parent the gene with the highest percent identity to the retrocopy. However, there were many cases in which retrocopies subsequently were copied via segmental duplications that occurred relatively recently during primate evolution [71,72]. To handle these cases, we selected the parental gene with the highest retrocopy score. Our aim here was not to unequivocally determine the correct parent gene among the segmentally duplicated copies, but merely to look for functional elements arising from retroposition regardless of whether they occurred before or after other duplications.

### Filtering out Zinc Finger, Mitrochondrial and Immunoglobin Genes

We found a high number of potentially false positives in our initial gene set due to the large size of the zinc finger, mitrochonrial and immunoglobin gene families. Since many of these copies were generated via mechanisms other than retroposition, we excluded all of these cases from our dataset.

### Determining Expression using mRNA and EST Evidence

The candidate retrocopies identified by the above process were then screened for an overlap with BLAT mRNA and EST alignments [64]. We used BLAT instead of BLASTZ since it is aware of splice sites and is better at aligning mRNAs to the genome. In cases where the retrocopy was very similar to the parent gene, we found that it was necessary to look at individual bases that were different between the two genomic locations. We required that the mRNA align better to the retrocopy location than did the parent gene at a minimum of 5 positions. We measured this by counting the number of sites (excluding SNPs) where an mRNA base differed from the genomic base to which it was aligned. A few such differences can occur in the alignment of the parent gene's mRNA to the DNA of the parent gene due to polymorphism, or to errors in the mRNA sequences [66]. If we could not uniquely identify the genomic locus for an mRNA or EST, then it was not considered evidence of expression. We used the program bestOrf [73] to score putative retrogenes for protein coding potential. Cases scoring less than 50 were removed. The program outputs potential CDS positions produced taking into account probabilities of each potential start codon, as well as longest ORF positions, extending the of CDS upstream from start codon). We then categorized the resulting set of expressed candidate retrogenes two ways: first, by type of evolutionary event, and second, by the strength of the evidence of expression. The entire pipeline is shown in Figure 3 (see additional files 4,5,6,7 for numbers) .

### Classification of Retrocopies according to Evolutionary Event

Finally, we classified the retrocopies found in our initial set of genes into three types. The Type I retrocopies contributed domains, in either the sense or antisense orientation, to known multiple coding exon genes [74] or to those found in the Refseq database [75]. Type II retrocopies consisted of duplications of one or more coding exons derived from the parent gene that formed new genes independent of host genes. Those cases with additional UTR exons or small coding exons derived from intergenic regions were also considered Type II. All remaining cases were considered Type III, defined as retrocopy contributions, in either the sense or antisense orientation, to novel genes out-of-frame with respect to the retrocopy parent, combined with major contributions from other types of retroposed elements and protein coding segments derived from the unannotated genomic environment. Retrogenes containing Type III retrocopies were defined as having no significant BLAST alignment to any other protein coding genes (using an e-value threshold of 0.01).

### Species Comparisons

In order to determine the age of the retrocopies, we used outgroup analysis. We checked for the presence and/or absense of all 726 candidate retrocopy genes in the following species (UCSC Genome browser databases * (shown in parenthesis) :Homo sapiens *(hg18), *Pan troglodytes *(panTro2), *Macaca mulatta *(rheMac2), *Mus musculus *(mm8), and *Canis familaris *(canFam2). For the recent events (based on their presence in human and rhesus monkey and absence in mouse and dog) we selectively looked at the trace archives of *Pongo pygmaeus abelii *(orangutan) and *Callithrix jacchus *(marmoset). We manually assembled traces orangutan and marmoset from the NCBI trace archive to determine if the reading frame was open in the cases shown in tables 2 and 3. In a few selected cases of older events (present in all of the above species), we also examined chicken (galGal3) and *C. elegans *(ce2).

## Authors' contributions

RB developed the algorithm, carried out the molecular genetic studies, performed the sequence alignment and drafted the manuscript. MD participated in the data analysis and methods development, JK participated in the sequence alignment and algorithm development, DH conceived of the study, participated in its design and edited the manuscript, JB participated in analysis of data, interpretation of the results and drafting of the manuscript. All authors read and approved the final manuscript.

## Supplementary Material

Additional file 1 **Entire list of 726 candidate expressed retrocopies with strong evidence.** Click here for file

Additional file 2 **Classes of Type II retrogenes.** Click here for file

Additional file 3 **Details on additional examples and Additional methods.** Click here for file

Additional file 4 **More novel Type III gene candidates.** Click here for file

Additional file 5 **Type II retrogenes – selected cases.** Click here for file

Additional file 6 **TXNDC2 chimeric retrocopy.** Click here for file

Additional file 7 **DGCR13 and TSSK2 alignments in multiple primates.** Click here for file
