# Supplementary material for: Retrocopy contributions to the evolution of the human genome
Source: BMC Genomics. 2008 Oct 8;9:466. doi: 10.1186/1471-2164-9-466 (PMC2584115; doi:10.1186/1471-2164-9-466)
Supplement: Additional file 2 — Classes of Type II retrogenes. [file 1471-2164-9-466-S2.pdf]

Supp. Table 2 - type II retrogenes - selected cases

Gene	Parent Gene	Fig	Evolutionary Event	Expresion Evidence	% in frame 1	% in frame 2	% in frame 3	Coding Bases Aligned	Total Bases Aligned	Late Start (bp)	Early End (bp)	Retro Exon Count	Retro Coding Exons
NACAP	NACA	S1-1	single exon	6 mRNAs	16.4	83.6	0	408	768	45	188	1	1
RANBP6	RANBP	S1-1	single exon, alt late intron	7 mRNAs, 5 ESTs	0	0	100	3277	3339	73	0	1	1
WDR40B	WDR40A	S1-2	3' UTR exons	1 spliced mRNA, 5 ESTs	0	3.8	96.2	1330	1463	276	11	2	1
RPL39L	RPL39	S1-2	5' UTR exons	4 spliced mRNA, > 10 spliced ESTs	100	0	0	95	382	579	0	3	1
LOC144983	HNRPA1	S1-2	5' UTR exons	5 spliced mRNA, >10 spliced EST	0	100	0	963	1340	0	0	7	1
JMJD2D	JMJD2C	S1-2	5' UTR exons	1 spliced mRNA, 10 spliced ESTs	0	0	100	1038	1038	0	0	3	1
RAB40A	RAB40B	S1-2	5' UTR exons	1 spliced mRNA , 3 spliced EST	0	100	0	779	1655	6151	41	3	1
USP29	USP37	S1-2	5' UTR exons	3 spliced mRNA, 6 spliced ESTs	0.1	99.9	0	2710	2859	601	1	4	1
NM_001013648	FUNDC2	S1-3	new 3' UTR exon	2 mRNAs (1 spliced), > 15 ESTs (1 spliced)	90.4	9.6	0	271	773	0	32	2	1
FAM113B	FAM113A	S1-4	early stop	4 spliced mRNA >10 spliced ESTs	4.8	0.5	94.7	1225	1404	46	4	4	1
PLEKHA9	PLEKHA8	S1-6	N terminus extended by	2 spliced mrna, 4 spliced EST	0	0	100	871	1397	0	437	3	1
MGC70863	RPL23A	S1-7	C terminus extended by parent UTR	1 spliced mRNA, >20 spliced ESTs	0	6.3	93.7	347	513	244	0	4	1
CDY1	CDYL	S1-8	internal intron	3 spliced mRNA	100	0	0	1620	2752	693	26	2	1
NUDT10	NUDT4	S1-10	late start	3 spliced mRNA, 15 spliced ESTs	1.1	98.9	0	542	1718	386	0	3	2
U2AF1L1	ZRSR2	S1-11	hijacks SRP19 promoter	1 spliced mRNA, 10 splced ESTs	1457	0	100	0	1422	0	0	4	2
HS6ST3	HS6ST2	S1-12	late intron	2 spliced mRNA, 1 spliced EST	0	4.7	95.3	450	714	2566	0	2	2
YWHAH and YWHAG	YWHAB	S1-12	late intron	9 spliced mRNA, >10 spliced ESTs	631	0	100	0	631	0	0	2	2
ARMCX1	ARMC10	S1-13	new coding sequence	7 spliced mRNA >20 spliced ESTs	1.4	96.8	1.8	843	1284	262	0	4	1
KLHL25	KLHL6	S1-14	frayed ends	8 spliced mRNAs	80.3	8.8	10.9	1460	1969	10891	0	3	1
