# Supplementary material for: Retrocopy contributions to the evolution of the human genome
Source: BMC Genomics. 2008 Oct 8;9:466. doi: 10.1186/1471-2164-9-466 (PMC2584115; doi:10.1186/1471-2164-9-466)
Supplement: Additional file 3 — Details on additional examples and Additional methods. [file 1471-2164-9-466-S3.doc]

**Additional Methods**

**Procedure for mapping of mRNA and ESTs**

We used the following commands for mapping mRNAs and ESTs to the genome for the following: 1) determine location of parent gene; 2) determine if the retrocopies are expressed.

mRNA Method:

blat -t=dna -q=rna -fine -ooc=11.occ -repeats=lower

Aligns RNA to the genome preventing alignment seeding on highly repetitive regions (11-mers) and repeats.

pslCDnaFilter -minId=0.95 -minCover=0.25 - globalNearBest=0.0025 -minQSize=20 -minNonRepSize=16 -ignoreNs -bestOverlap -polyASizes=mrna.polya

We use the same filters that are employed by the UCSC Genome Browser to place mRNAs and ESTs. pslCDnaFilter filters the BLAT results to remove hits that are: 1) lower than 95% identical; 2) cover less than 25% of the RNA (excluding the poly(A) tail); and 3) requires at least 20 bases (16 bp must be non-repetitive) of the RNA to align. If there are multiple hits for the same mRNA, the globalNearBest option throws out the second best hit if the alignment score is greater than a relative threshold compared with the best scoring region. The alignment score is based on percent identity and adds a bonus for multiple exons and a penalty for insertions or deletions. 0.25% (fewer than 1 in 400 mismatches) was chosen to exclude pseudogenes and paralogs but keep regions that are so recently duplicated where sequencing errors overwhelm the natural mutation rate.

EST Method:

blat -ooc=11.occ -repeats=lower

pslCDnaFilter -minId=0.95 -minCover=0.25 -globalNearBest=0.0025 -minQSize=20 -minNonRepSize=16 -ignoreNs -bestOverlap -polyASizes=est.polya –usePolyTHead

The EST method is similar but also excludes poly(T) tails since we are not sure of the orientation of ESTs.

**Supplemental Text**

# Type II duplication events

The most common type of retrogene is the simple duplication event generating a single exon gene [See Additional File 2, category 1 and Additional File 5]. Since the late eighties, there has been a constant stream of discoveries of functional retrogenes (reviewed by Brosius, 1999a) providing dozens of additional predicted cases ranging from ancient to more recent events (Harrison et al. 2005; Vinckenbosch et al. 2006). Of the Type II events revealed in our screen, a large number contained one or more 5’ and/or 3’ untranslated exons that were acquired from the flanking regions of the insertion loci. New acquisitions of distal regulatory regions were often followed by intronization of large parts of the UTR regions. [See Additional File 4, categories 2 and 3], an event that was predicted previously (Brosius and Gould, 1992; Brosius 1999b). In addition to 245 cases reported previously (Vinckenbosch et al. 2006), we provide a total of 714 cases whose integrations are both ancient and “recent” (exclusive to primates) [See Additional File 6].

There are cases in which the original ORF was truncated due to mutations that led to earlier stop codons - fraying of the termini of the potential protein. For example, *FAM113B* [See Additional File 2, category 4] a *FAM113A*-derived retrocopy acquired a 5’ UTR exon from the flanking sequences and would encode a shortened C-terminal due to an in-frame stop codon. The gene is conserved in mammals. An analogous situation is conceivable in the N-terminals encoded by retrogenes, when the start codon was lost and the gene recruited a later start codon from the protein coding region [See Additional File 2, category 5].

Likewise, *extensions* of the hypothetical protein terminals can occur by several mechanisms. One possibility is the acquisition of triplet codon sequences out of 5’ or 3’ UTRs by acquisition of earlier start codons or later stop codons, respectively. For example, *PLEKHA9* [See Additional File 2, category 6] features an elongated N-terminal encoding exon. The start codon was derived from the 5’ UTR of the retrocopy, and the stop codon from the parent. *PLEKHA9* was inserted into the ape lineage after divergence from the Old World monkey branch and shares a bi-directional promoter with *TMEM16F*. However, the gene does not appear to be under strong selection, as the ORF in chimpanzee is disrupted by a frameshift and a subsequent stop codon.

*MGC70863* is an *RPL23a*-derived retrocopy with a later start codon [See Additional File 2, category 7]. A one base pair deletion in the C-terminus skips the original parent gene-derived stop codon and extends the ORF by 7 triplet codons generating an ORF of 121 codons. The retrocopy is present in rhesus monkey but is not under selection or only weak purifying selection as the gene appears not to persist: the rhesus monkey features an in-frame stop codon due to an indel, truncating the hypothetical protein after 20 aa, and the chimpanzee has a sequencing gap at the orthologous position.

**“Late” Introns**

Some examples, in which introns arose in flanking UTRs subsequent to retrocopy insertion, have been reported recently (Vinckenbosch et al. 2006). We found no indication that such sequences were transcribed introns prior to the insertion of the retrocopy. Occasionally, we observed that a 5’ or 3’ exon recruited from the locus provided the first or last coding exons, in addition to the UTR (see below). This underscores the notion that intron-containing genes, especially those with large exons, cannot be excluded from having had a retroposition origin.

We also identified the single exon *CDY1* gene on the Y chromosome, reported by Lahn and Page (1999) to be a *CDYL*-derived retrogene from chromosome 6. In addition to the major unspliced transcript, a minor splice variant is described that probably was facilitated by a point mutation close to the splice site (Lahn and Page, 1999). In this variant the C-terminal encoding 9 triplet codons (also corresponding to the same in the *CDYL* parent) are skipped. However, 23 new C-terminal codons were derived from the retrogene’s 3’ UTR that also coincides with the 3’ UTR of the parent gene. In other words, the fortuitous acquisition of weak splice sites generated an intron between the C-terminal part of the ORF and the 3’ UTR, making part of the 3' UTR a second protein coding exon [See Additional File 2, category 8]. The *CDY1* retrogene arose either prior to or shortly after the primate diversification (Dorus et al., 2003). The answer to when the minor transcript arose awaits additional primate sequences of chrY and perhaps experimental confirmation of the splice form in various primates.

It is conceivable that retrogenes exist in which novel introns were generated exclusively in their ORFs that also correspond to the ORF, but of course not to the splice sites, of the parent gene. This would be possible when both donor and acceptor splice sites arose in the ORF of the retrocopy [See Additional File 2, category 9], Another scenario would place the donor splice site in the ORF of the retrocopy and the acceptor beyond the retrocopy (e.g., from intergenic sequences). One such example of a retrogene with protein coding sequence from the flanking sequence is *NUDT10* [See Additional File 2, category 10] that was inserted on ChrX and acquired a 5’ UTR exon and a 3' coding exon from the sequence flanking the insertion. In the *NUDT10* example, the 3’ coding exon happens to have a single codon that is a stop codon as well as a long UTR. The retrogene (164 triplet codons) is shorter than the parent *NUDT4* (180 triplet codons) because exonic sequences were lost when a “late intron”, arose in the 3’ end of the ORF and is spliced onto the 3’ coding exon. *NUDT10* is conserved in mouse, dog, and rhesus monkey. An analogous situation *U2AFIL1* that involves recruitment of the N-terminal protein coding exon from *SRP19* is shown in Additional File 2, category 11.

We also observed cases where intronic sequences that interrupt the retrogene (in what corresponds to the ORF of the parent gene) apparently are not derived from the retrocopy and whose origin is still unclear, for example, *HS6ST3* [See Additional File 2, category 12]. The parent gene *HS6ST2* (644 triplet codons) has 8 exons, and led to a new retrogene that currently has 2 coding exons comprising 471 triplet codons. The orthologous mouse gene, *hs6st3*, also has two exons - so this is a relatively ancient event.

We found another interesting example of apparent intron acquisition interrupting the ORF of the retrogene [See Additional File 2, category 12]. *YWHAG* (derived from *YWHAB*) gained an intron that is also present in retrogene *YWHAH*. It is noteworthy that the position of the intron is different from any that are present in the presumed parent gene. “Parenthood” is somewhat complicated by the fact that in humans there are four genes (*YWHAB*, *YWHAZ*, *YWHAE*, and *YWHAQ*), each of which harbor five exons (*YWHAE* has six) in the protein coding region. At some point, the extra copies must have arisen by segmental duplication or whole genome duplication. Due to its high degree of sequence similarity, we assume that *YWHAB* spawned the retrogene *YWHAH*. The latter covers all of the exons (no corresponding introns) of the parent gene and has been preserved from fish to mammals. After the retroposition event, *YWHAG* probably was derived from *YWHAH*, or vice versa, by segmental duplication. In any event, the origin of this large and “relatively late” intron (28 kb and 11 kb in *YWHAG* and *YWHAH*, respectively) between codons 28 and 29 is enigmatic. One explanation is that after retroposition, but prior to its segmental duplication, the retrogene acquired an internal intron somewhere during diversification of vertebrates. Chicken also has a *YWHAH* gene (chr15) with a single intron, precisely at the same position as mammalian *YWHAH*. The divergence of the previously acquired intron in mammalian *YWHAG* and *YWHAH* could be explained by a relatively early segmental duplication event on the lineage leading to mammals such that a possible relationship between the neutrally evolving introns became indiscernible. In addition, there is a truly intronless gene (*SFN*) in humans that might be orthologous to an intronless chicken gene (*SRCRB4D*); we did not find a similar, completely intronless gene in fish.

Several members of this gene family have spawned about 20 additional transcribed as well as untranscribed retrocopies. The expressed retrogenes *YWHAG* and *YWHAH* are not present in flies, which have two *YWHA*-related copies (epsilon and zeta) with 4 and 5 (+ 3 alternatively spliced) exons in the ORF, respectively. Only one intron position in both paralogs is conserved in vertebrates. In *C. elegans*, there are three related genes: *Ftt2* shares 3 out of 4 ORF introns with mammalian *YWHAB*; *Par5* contains 3 introns and *M117.3* matches *Par5*, but is 5’ truncated and shares only the last intron with *Par5*, but all are at different positions than in Ftt2 or in any of the vertebrate homologs, except for the first Par5 intron that precisely matches the position of vertebrate *YWHAG* and *YWHAH*. Therefore, we also have to consider an intron-loss instead of an intron-gain scenario, where *YWHAG/H* arose in the lineage leading to vertebrates from a *Par5*-like gene by partial intron loss (presumably by recombination with a retrocopy, see below) except for the remaining intron which was lost in one or several invertebrate branches but persisted in vertebrates.

A somewhat analogous example involving the acquisition of an exonic sequence from an unknown source is documented in Additional File 2, category 13. ARMCX1 is devoid of introns in the ORF (three 5’ UTR exons were recruited out of flanking sequences) and was presumably derived from the SVH parent gene by retroposition in a common ancestor of placental mammals (opossum lacks ARMCX1). The translational start and stop codons coincide with those of the parent gene. However, human ARMCX1 and other mammalian orthologs contain an insertion (encoding 168 aa in human) in the ORF after codon 30 of the SVH/ARMC10 ORF. The mystery is that, thus far, there is no indication as to the origin of the extra sequence, except for a weak hit (75bp) to a LINE element. BLASTZ and protein searches revealed no similarity to any sequence other than the aforementioned orthologs in placental mammals. One possible explanation is that an alternative form of the parent gene existed, which included an additional exon, that has since been lost, or it could be a copy of DNA from an another unsequenced part of the genome (e.g., paracentromeric region). These examples are evidence that the presence of introns or exonic inserts do not exclude a retropositional origin of genes. For a review on recent intron acqusition see Roy (2004).

*KLHL25* is a *KLHL6*-derived retrogene that goes in and out of frame many times but nevertheless has multiples-of-three indels to maintain part of the frame [See Additional File 2, category 14]. It acquired a 5' and 3' UTR exon from the insertion locus and has frayed ends so that start and stop codons do not exactly correspond to those of the parent. The gene is conserved in mouse and dog. This example leads to our Type III category where we observe the contribution from the parent gene via the retrocopy using little of existing protein sequence space.

# Additional Type III Novel Candidate genes with contributions of retrocopies

*TXN* is a parent of retrogene *TXNDC2*, which would encode 104 out of 105 aa of the parent gene except for the N-terminal methionine, as the coding region of *TXNDC2* extends further more N-terminally, for a total of 553 aa. The stop codon, however, is shared with the parent [See Additional File 4, example A]. What are the remaining 381 aa coded from? Eighty-nine triplet codons at the N-terminal encoding region do not align to any known sequence and appear to have been recruited from an unknown source. The center of the ORF consists of 23 more or less degenerate retrocopies, usually of 45 bp (each encoding 15 aa) (Figure S3). The protein repeat domain aligns to the titin (TTN) protein (e-value 3x10^-23^), featuring many repeats and contains a tandem Ig cluster (Radke et al. 2007). The C-terminus encoding part of *TXNDC2* does not align to *TTN*, and thus, the novel gene is composed of two fused retrocopies with the N-terminus encoding region exapted from an unknown source, presumably the locus of integration. The *TXNDC2* ORF is open in dog, mouse, rhesus monkey, and human, but not in chicken, and we did not find it in platypus or opossum. This suggests that the *TXN* retrocopy was appended to a retrocopy of a portion of an Ig cluster early in mammalian evolution or slightly before. More genome sequences are necessary to accurately date the fusion event. Interestingly, there is another gene, *PRAM1*, that has a retroposed portion of *TTN* from the same region as does *TXNDC2* that forms a very large exon. The other 9 exons are very small and do not align to *TTN*, but exhibit sequence similarity to a kinase encoding domain. Although the *TTN* gene also contains a kinase domain, its large size (encoding 33,423 aa) makes it difficult to be certain that *PRAM1* (encoding 669 aa) is a shorter paralog. A likely possibility is that, somewhat analogous to *TXNDC2*, the TTN-derived portion (encoding 552 aa) was recruited into a pre-existing gene that contained a kinase domain, hence a type Ia situation. The events involving *PRAM1* and *TXNDC2* are not young as both feature an ORF in mouse and dog. Once more, incomplete sequences in the more distant mammals make reconstruction of events difficult without further information.

The intron-containing parent gene *CDC14B* yielded a retrocopy in primates prior to the branching of New World monkeys, which was followed by the integration of a MER9 LTR element into the C-terminus encoding part of what corresponds to the ORF of the parent gene. A segmental duplication prior to the branching of apes then yielded a second copy. Thereafter, one of the copies was interrupted by insertion of a truncated L1PA3 LINE element [See Additional File 4, example B]. There is no expression evidence for the sense orientation of the copy with the LINE element. The active gene candidate, *AK127327* is in the opposite orientation to the retrocopy and consists of a 5’ UTR and an N-terminal encoding portion of the ORF contributed by the ORF of the retrocopy. The C-terminal encoding portion of the *AK127327* ORF is contributed by the 5’ UTR of the retrocopy. The *AK127327* ORF continues for 6 aa into the unannotated sequence of the insertion locus. Unannotated sequences also contribute the 3’ UTR of *AK127327* [See Additional File 4, example B]. Human and chimpanzee have an open ORF encoding 136 aa; orangutan has a C-terminal extension yielding an ORF encoding 152 aa. Rhesus monkey has a single retrocopy (without LINE insertion) and the antisense ORF is open and encodes 144 aa at this similar but non-orthologous locus. The position of the start codon is conserved in human, chimpanzee and rhesus monkey.

Another idiosyncratic Type III case to be introduced in detail involves a retrocopy derived from parent *DFFB* (6 introns), which is upstream from the *TOPORS* gene [See Additional File 4, example C]. Two transcripts are generated from that locus in the antisense orientation to the two aforementioned genes. The first transcript (nalee.cAug05) begins with a 5’ UTR and first protein coding exon in the first intron of the *TOPORS* gene (antisense). This first exon is spliced onto a second exon that overlaps the short first protein coding exon of *TOPORS*. The third exon overlaps with the 3’ UTR of the retrocopy (antisense). Expression of this transcript is supported by one mRNA and 20 spliced ESTs. The second transcript (*FLJ25547*), supported by one mRNA and 4 spliced ESTs, originates in a region overlapping the second exon of nalee.cAug05, but it is 5’ untranslated. The splice leads onto the retrocopy to what corresponds to the ORF of the parent gene in antisense. This region still contributes to the 5’ UTR of *FLJ25547*. In an area that corresponds to the N-terminus encoding exon of the retrocopy’s parent, the ORF starts and leads into an area proximal to the retrocopy consisting of a tigger DNA transposon that, in turn, is interrupted by two Alu elements. This is where translation terminates after encoding 195 amino acids [See Additional File 4, example C]. In chimpanzee, nalee.cAug05 is not feasible, but *FLJ25547* contains the ORF with 3 amino acid replacements. In orangutan the ORFs of both forms are truncated: the hypothetical protein encoded by *FLJ25547* lacks 81 amino acids at the C-terminus.

The next case is represented by *FLJ45974*, where an LTR contributes the first exon including the start codon and 139 triplet codons that continue into the unannotated sequence of gene locus [See Additional File 4, example D]. The second exon recruits the next part of the ORF, 31 codons out of a retrocopy (derived from parent *RAC1*) in the antisense orientation and the final coding exon covering 4 codons and the stop codon is from unannotated sequences (the entire ORF would encode 174 aa). The proximal part of the 3’ UTR is still out of unannotated sequences, and continues into a truncated L1MC1 LINE element. Three additional 3’ UTR exons are recruited from this and an adjacent L1MCA LINE element as well from flanking sequences [See Additional File 4, example D]. The ORF is not conserved in chimpanzee (several frame shifts, leading to translation stops). In orangutan the combined reading frame of the exons is open and C-terminally extends for an unknown stretch (sequence gap) and on paper, the splice sites are present (the donor of exon 1 is shifted by one triplet).

A highly unusual putative retrogene is presented in Figure S2, example E: The parent gene *TSSK4* (4 exons) gave rise to retrogene *TSSK2* (row ii), transcribed in the same orientation as TSSK4 (row i). At first sight, it is similar to a Type II retrogene [See Additional File 2, category 4] formed from exons 1-4 of *TSSK4* comprised of about 290 triplet codons from the parent followed by about 68 codons from intergenic sequences (due to the age of the retrocopy, the precise borders cannot be established). Despite the mammalian conservation of *TSSK2*, the sequence of the orangutan locus revealed a one bp insertion resulting, somewhat downstream, in a stop codon, 62 triplet codons from the 3’ ORF of the human *TSSK2* orthologue. EST evidence indicates additional transcription in the antisense orientation and, surprisingly, in the human locus the opposite reading frame is open as well (encoding 339 aa), starting slightly before the stop codon and ending somewhat before the start codon of *TSSK2* – but in the reverse orientation. A gene, *DGCR13* in the opposite orientation [See Additional File 4, example E, row iii] has been described (Gong et al. 1996) without the realization that the ORF is almost precisely on the opposite strand of the one encoding the *TSSK2* protein. There is a possibility that *DGCR13* (recently retracted from NCBI Refseq) is a long UTR of the nearby *DGCR14* gene. While numerous overlapping genes (Finta and Zaphiropoulos 2000) are known in the human genome (reviewed in Makalowska et al. 2005), such an extensive antisense arrangement has not been reported in Eukarya. The *TSSK2* gene is conserved at least in mammals. Gibbon, rhesus and marmoset have rather early stop codons in the *DGCR13* direction, gibbon even lacks an ATG start codon [See Additional File 7]. In orangutan, the aforementioned one bp insertion resulting in a slight truncation of the *TSSK2*-encoded C-terminus, results in a frameshift and a concomitant early truncation of the *DGCR13*-encoded protein after 79 aa. Only three great apes (human, chimpanzee and gorilla) harbor a full *DGCR13* ORF [See Additional File 7]. This ORF could have arisen fortuitously early in primate or even mammalian evolution, but selection pressure was never high enough to prevent loss in one or the other lineage. The ORF might have (re)opened after gorilla, chimpanzee, and man split from the other apes and now might be under weak selection. Alternatively, the *DGCR13* ORF may have opened fortuitously in a common ancestor of gorilla, chimpanzee and human and persisted for >8-10 million years because of some weak negative selection or just by chance, because the opposite *TSSK2* ORF is under stronger selection .

Brosius J, Gould SJ: **On "genomenclature": a comprehensive (and respectful) taxonomy for pseudogenes and other "junk DNA".** *Proc Natl Acad Sci* 1992, **89:**10706-10710.

Brosius J, Tiedge, H: **Reverse transcriptase: mediator of genomic plasticity*.*** *Virus Genes* 1995, **11:**163-179.

Brosius J: **Genomes were forged by massive bombardments with retroelements and retrosequences.** *Genetica* 1999a, **107:**209-238.

Brosius J: **Many G-protein-coupled receptors are encoded by retrogenes.** *Trends Genet* 1999b, **15:**304-305.

Dorus S, Gilbert SL, Forster ML, Barndt, RJ, Lahn BT: **The CDY-related gene family: coordinated evolution in copy number, expression profile and protein sequence.** *Hum. Mol. Genet* 2003, **12:** 1643-1650.

Dupuy D, Duperat VG, Arveiler B: **SCAN domain-containing 2 gene (SCAND2) is a novel nuclear protein derived from the zinc finger family by exon shuffling.** *Gene* 2002, **289:**1-6.

Gong W, Emanuel BS, Collins J, Kim DH, Wang Z, Chen F, Zhang G, Roe B, Budarf ML: **A transcription map of the DiGeorge and velo-cardio-facial syndrome minimal critical region on 22q11.** *Hum. Mol. Genet* 1996, **5:** 789-800.

Finta C, Zaphiropoulos PG: **The human cytochrome P450 3A locus. Gene evolution by capture of downstream exons.** *Gene* 2000, **260:**13-23.

Hao, Z, Jha, KN, Kim, YH, Vemuganti, S, Westbrook, VA, Chertihin, O, Markgraf, K, Flickinger, CJ, Coppola, M, Herr, JC, Visconti, PE: **Expression analysis of the human testis-specific serine/threonine kinase (TSSK) homologues. A TSSK member is present in the equatorial segment of human sperm.** *Mol Hum Reprod* 2004, **10:**433-444.

Harrison PM, Zheng D, Zhang Z, Carriero N, Gerstein M: **Transcribed processed pseudogenes in the human genome: An intermediate form of expressed retrosequence lacking protein-coding ability.** *Nucleic Acids Res* 2005, **33:** 2374–2383.

Kent WJ: **BLAT--the BLAST-like alignment tool**. *Genome Res* 2002, **12**:656-664.

Makalowska I, Lin CF, Makalowski W: **Overlapping genes in vertebrate genomes.** *Comput Biol Chem* 2005, **29:**1-12.

Radke MH, Peng J, Wu Y, McNabb M, Nelson OL, Granzier H, Gotthardt M: **Targeted deletion of titin N2B region leads to diastolic dysfunction and cardiac atrophy.** *Proc Natl Acad Sci U S A* 2007, **104:**3444-3449.

Roy, WR: **The origin of recent introns: transposons?** *Genome Biology* 2004, **5:**251.

Vinckenbosch N, Dupanloup I, Kaessmann H: **Evolutionary fate of retroposed gene copies in the human genome.** *Proc Natl Acad Sci U S A* 2006, **103:**3220-5.

**Additional File 1 – all retrocopy examples.** All 726 retrocopies with strong expression evidence (See Methods) are listed. The columns are defined as follows Chrom, chromStart, chromEnd – genomic coordinates of retrogene, name – unique id of retrogene based on parent mRNA accession, score – retrogene score of combined features described in methods, strand – orientation of retrogene, axtScore – chained blastz score of parent gene aligned to retro, type – expression evidence, parent chrom, parent start, parent end, parent strand - genomic location of parent gene, parentSpliceCount – number of splice sites in parent gene, poly(A) distance from 3' end, exonCover- number of parent exons that align to retrogene, intronCount – number of introns in retrogene, bases matching parent – number of identical bases in alignment from retro to parent gene, mRNA Size – bases in parent mRNA, bases from 3' end – number of bases truncated at 3’ end of alignment, %Repeats – percent coverage of retrogene that overlaps RepeatMasker, %break in orthology rhesus – percent coverage of retrogene not orthologous to rhesus monkey, %break in orthology mouse, %coverage – fraction of parent gene covered by alignment, %identity (times ten) with parent, processedIntrons – number of introns spliced out from retrocopy, conservedSpliceSites – number of parent splice sites that align to retrocopy, bases Overlapping mRNA – number of bases in retrocopy overlapping any spliced mRNA, refSeq parent - refseq accession of parent, mgc parent, mgc start mgc end, known Gene parent, overlapping mRNA- accession of overlapping mRNA, %break in orthology dog, poly(A) length – length of poly(A) tail at retro locus

**Additional File 2.** **Categories of type II retrogenes defined by contribution of parent gene and other acquired features.** Not drawn to scale, splice events are marked by angled black lines, open reading frames are depicted as vertically striped thick bars, UTRs by medium size bars, introns by thin lines, promoters as arrowheads (see also color key in Fig. 2). 1) new gene variant derived from the parent gene (magenta) fused to promoter and 5’ UTR recruited from locus of integration (blue). 2) New splice sites arisen in the 5’ UTR of the sequence; UTR exons not present in parent gene. 3) New splice sites arose in the 3’ UTR of the retrogene. 4) In-frame stop codon toward the end of the retrogene results in a shortened ORF. 5) ORF shortened in the retrogene by use of a later start codon. Some of the retrocopy’s ORF turned UTR (dark red medium size bar) 6) longer ORF by recruiting additional protein coding sequence from the 5’ UTR of the parent gene (dark red). 7) ORF longer in the retrogene by recruiting additional protein coding sequence from the 3’ UTR of the parent gene. 8) Acquisition of an alternatively spliced coding exon from the 3’ UTR sequence. 9) Intron added to retrogene by adding splice sites in what used to be the ORF in the parent mRNA, resulting in the loss of protein coding sequence. 10) ORF shortened at N-terminal encoding exon by late start codon and C-terminus exchanged by new protein coding exon derived from flanking DNA. 11) First protein coding exon (light blue) recruited from an existing gene at the locus of integration. 12) Intron added to retrogene where new intron is not alignable to the genome due to deletion or divergence. 13). Acquisition of protein coding sequence from unknown source. 14) Mostly in-frame duplication with some new protein sequence generated out-of-frame with respect to parent.

**Additional File 3. Supplementary Examples and Methods**

**Additional File 4. Additional novel protein-sequence space combined with other transposons or unusual events (type III).** For each part of the figure (B-E), the spliced parent mRNA is shown first (before retroposition) and the resulting retrogene shown below. New sequence space was generated by a combination of retrocopy insertions, recruitment of non-genic regions including retroposons, whereby the contribution of the retrocopy’s original in-frame ORF is very small [See Addiional File 3 and legend to Fig. 1 including color key for further details]. A) Two different isoforms of *TXNDC2* formed from two fused retrocopies are shown. The N-terminal coding portion arose from the flanking unanntotated region (blue) and the two *TTN* and *TXN*-derived portions (magenta) are shown separated by a black bar. See Additional File 6 for the protein domains including the repeat structure of the TTN-derived sequence. B) Novel candidate gene *AK127237* generated from a CDC14B-derived retrocopy in the opposite orientation. More recent transposons interrupt the retrocopy. C) Two different novel gene candidates are generated from a *DFFB*-derived retrocopy in combination with Alu and Tigger insertions, an anti-sense 5’ portion of the *TOPORS* gene, and flanking DNA. D) Novel gene candidate *FLJ45974* is generated from a *RAC1*-derived retrocopy inserted in the antisense orientation and an LTR. The final coding exon is generated out of flanking DNA. E) *MARK4*-derived retrogene generates two ORFs in opposite orientation in the same genomic locus: *TSSK2* that is conserved in mammals, and an ape-specific novel anti-sense transcript *DGCR13* that may or may not code for protein [See Additional File 7].

**Additional File 5 - Type II retrogenes - selected cases.** List of example from type II retrogenes showing various types of modifications after insertion of the retrocopy. % in frame shows the fraction of bases in each of the three frames. Coding bases aligned is the number of coding bases in the retrogene that can be aligned to the parent gene. Total bases aligned shows all bases in the retrogene that align to the parent. Late start/early stop show the number of bases in the retrogene that are truncated with respect to the parent. Retro exon count is the number of exons in the retrocopy.

**Additional File 6.** **Amino acid sequence of the hypothetical protein encoded by the double retrogene *TXNDC2* in human.** A) The N-terminal portion (blue) does not align to other known proteins. The center section (black) is encoded from the retroposed TTN Ig domain. The encoded repeats are written underneath each other. The C-terminal portion (pink) is encoded by a retrocopy of *TXN*. B) Alignment of the C-terminal portion of TXNDC2 and the parent protein TXN. C) Alignment of the human TXNDC2 and TTN genes.

**Additional File 7 Protein and nucleotide sequence alignments of two open reading frames in the opposite orientation from several anthropoid primates.** TSSK2 (iii and iv, below) is a bona fide protein and gene, while DGCR13 (i and ii) is a hypothetical protein and gene that was removed from NCBI RefSeq. The sequences of human (*Homo sapiens*) and chimpanzee (*Pan troglodyes*) were downloaded from the UCSC Genome Browser hg18 and panTro2, respectively. Trace sequences of marmoset (*Callithrix jacchus*) were downloaded from the NCBI Trace repository and were assembled to yield a contig of this locus. This sequence probably contains several errors. We sequenced the remaining ape loci from gorilla (*Gorilla gorilla*), orangutan (*Pongo pygmaeus*) and gibbon (*Hylobates lar*). An asterisk below an alignment column indicates 100% identity.

iii) The amino acid sequence of TSSK2 shows a large degree of conservation, except for the orangutan locus that lacks 63 amino acids at the C-terminus. ii) Nucleic acid sequence alignment for the DGCR13 locus. The translational start and stop codons are highlighted in green and red, respectively. A one bp insertion in orangutan, leading to a premature stop is shown in magenta. The start and stop codons for DGCR13 in the opposite orientation are highlighted in grey. ii) The amino acid sequence of DGCR13 also shows a large degree of conservation, however, the ORFs of rhesus monkey, gibbon, and orangutan are very short, due to early stop codons (see part i). The potential start codons are highlighted in green. Due to a point mutation, gibbon is lacking an ATG start codon. The stop codons are highlighted in red and the one base insertion in orangutan in magenta. ii) Nucleic acid sequence alignment for the TSSK2 locus. The translational start and stop codons are highlighted in green and red, respectively. A one bp insertion in orangutan, leading to a premature stop is shown in magenta.
