# Supplementary figures and images for: Retrocopy contributions to the evolution of the human genome
Source: BMC Genomics. 2008 Oct 8;9:466. doi: 10.1186/1471-2164-9-466 (PMC2584115; doi:10.1186/1471-2164-9-466)

Suppl. Fig. 2    Novel Candidate Genes

A)

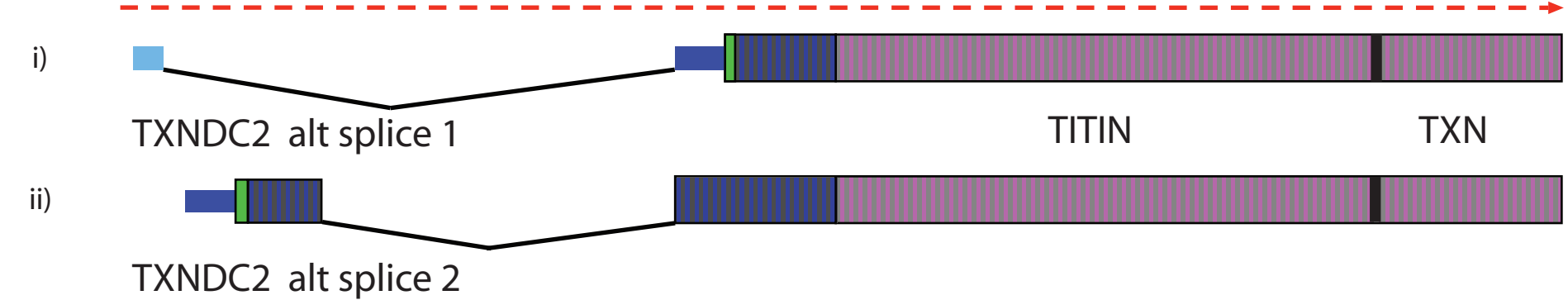

B)

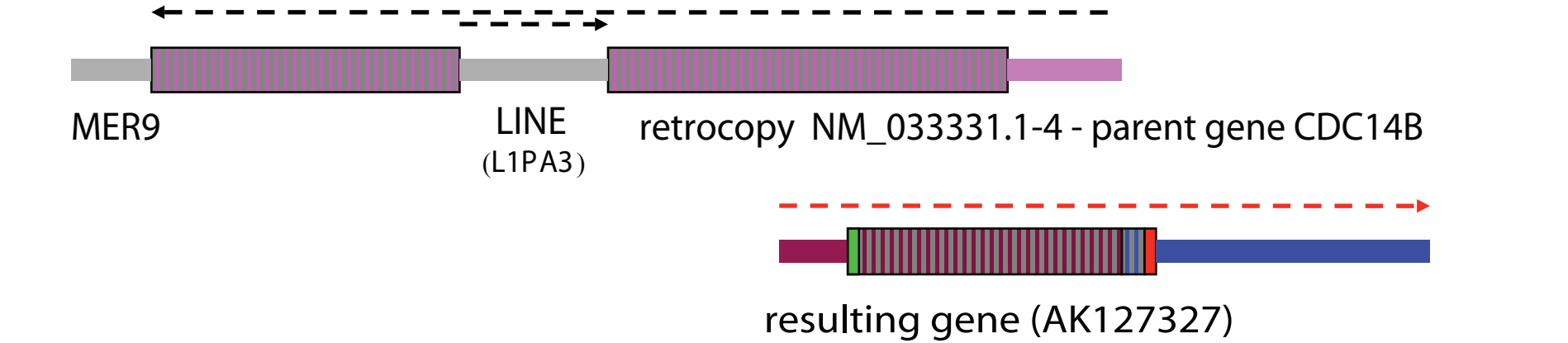

C)

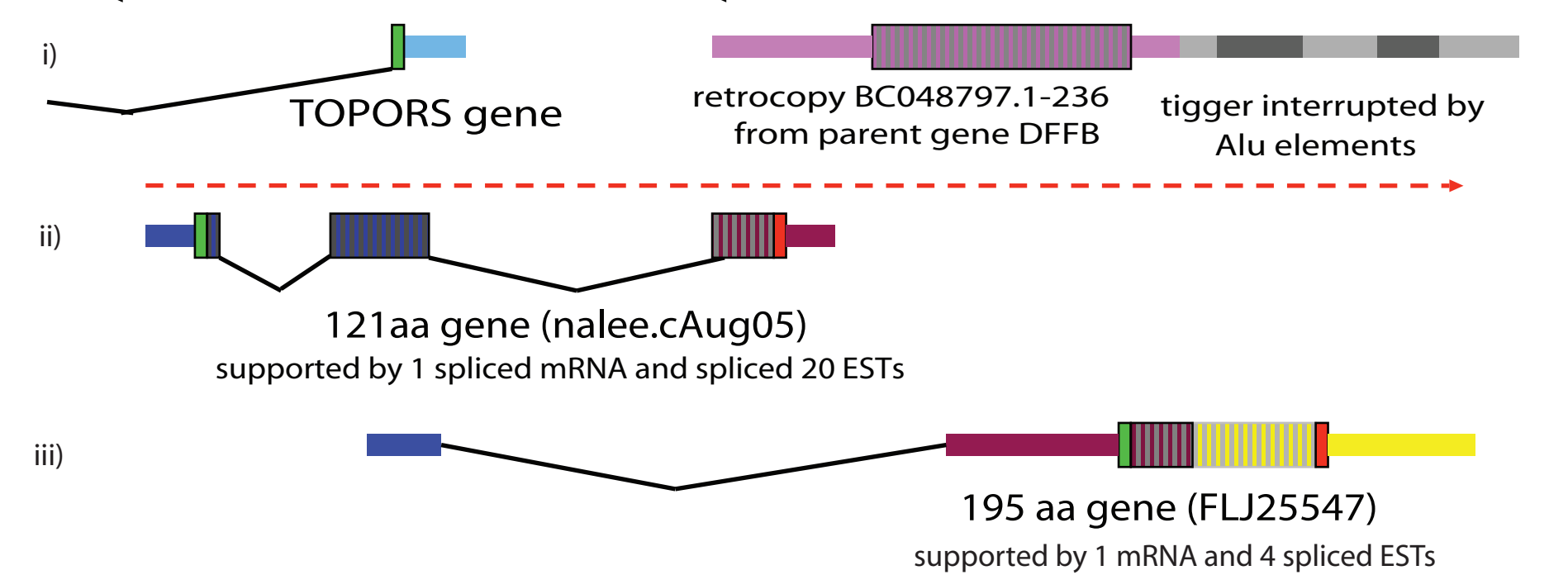

D)

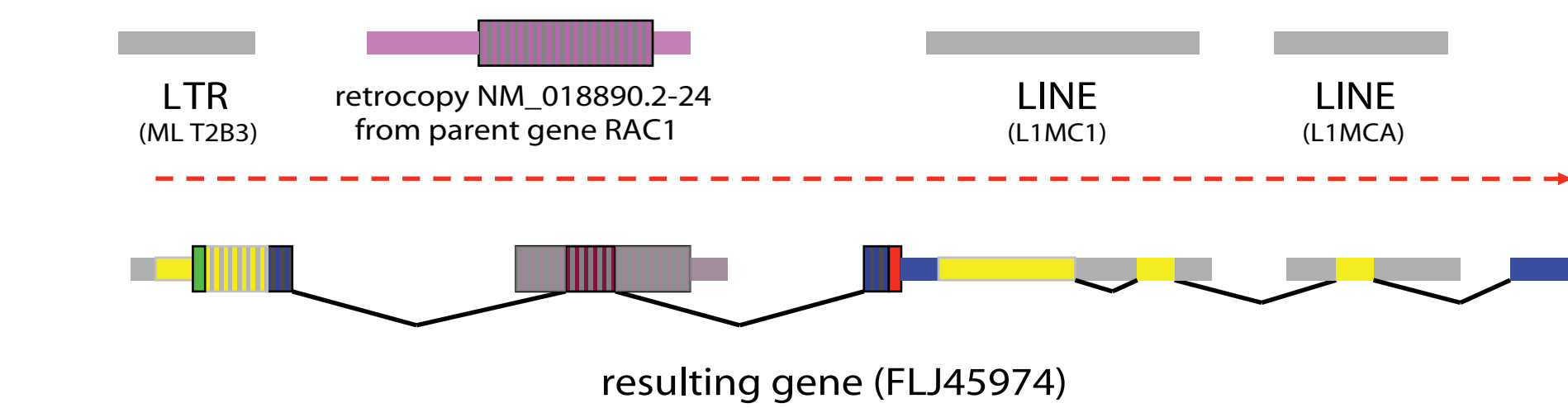

E)

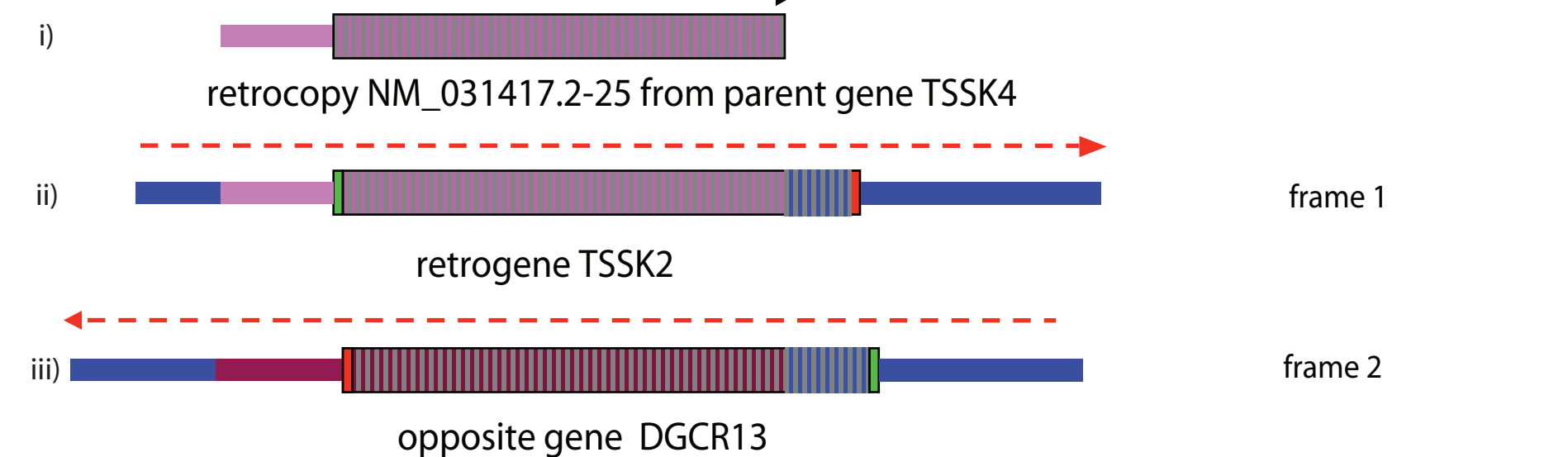

Supplement: Additional file 4 — More novel Type III gene candidates. [file 1471-2164-9-466-S4.pdf]

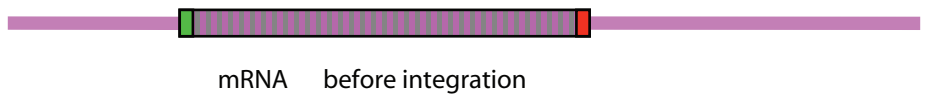

Suppl Fig 1 - type II

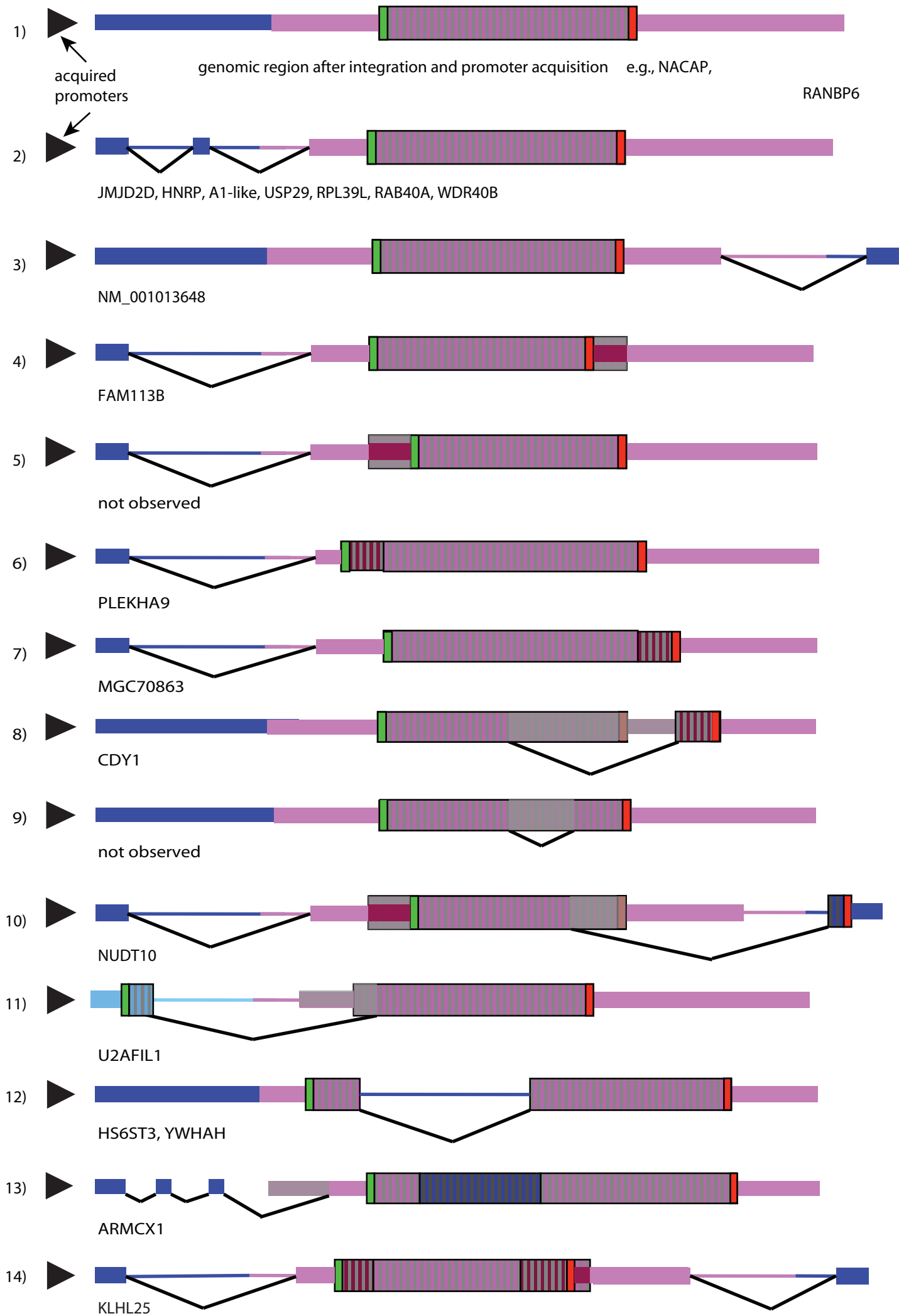

Supplement: Additional file 5 — Type II retrogenes – selected cases. [file 1471-2164-9-466-S5.pdf]
