# Supplementary material for: Retrocopy contributions to the evolution of the human genome
Source: BMC Genomics. 2008 Oct 8;9:466. doi: 10.1186/1471-2164-9-466 (PMC2584115; doi:10.1186/1471-2164-9-466)
Supplement: Additional file 6 — TXNDC2 chimeric retrocopy. [file 1471-2164-9-466-S6.pdf]

Supplemental Figure 3

A)

```
>TXNDC2_AK097656
MDVDKELGME SVKAGASGKP EMRLGTQEET SEGDANESSL LVLSSNVPLL
ALEFLEIAQA KEKAFLPMVS HTFHMRTEES DASQEGDDL
PKSSANTSHPKQDDS
PKSSEETIQPKEGDI
PKAPEETIQSKKEDL
PKSSEKAIQPKESNI
PKSSAKPIQPKLGNI
PKASVKPSQPKEGDI
PKAPEETIQSKKEDL
PKSSEEAIQPKEGDI
PKSSAKPIQPKLGNIAKTSVKPSQ
PKESDIPKSPEETIQ
PKEGDIPKSSAKPIQ
PKLGNIPKASVKPSQ
PKEGDISKSPEEAIQ
PKEGDLPKSLEEAIQ
PKEGDIPKSPEEAIQ
PKEGDIPKSLEEAIQ
PKKGDIPKSPEETIQ
PKKDDIPKSPEEAIQ
PKEGDIPKSPKQAIQ
PKEGDIPKSLEEAIP
PKEIDIPKSPEETIQ
PKEDDSPKSLEEAT
PSKEGDILKPEEETMEFPEGD
txn parent start
KVKVILSKED FEASLKEAGE RLVAVDFSAT WCGPCRTIRP FFHALSVKHE
DVVFLEVDAD NCEEVVRECA IMCVPTFQFY KKEEKVDELC GALKEKLEAV
IAELK
```

B)

Alignment between TXNDC2 (top uc002koh.1 486aa) and TXN (bottom uc004bep.1 105aa) score 6574

```
383 VKVILSKEDFEASLKEAGERLVAVDFSATWCGPCRTIRPFFHALSVKHEDVVFLEVDADN 442
    || | ||  |+ +|  ||++|| |||||||||||+ |+||||+|| |+ +|+||||| |+
002 VKQIESKTAFQEALDAAGDKLVVVDFSATWCGPCKMIKPFFHSLSEKYSNVIFLEVDVDD 061

443 CEEVVRECAIMCVPTFQFYKKEEKVDELCGALKEKLEAVIAEL 485
    |++|  || + |+|||||+|| +|| |  || |||||| | ||
062 CQDVASECEVKCMPTFQFFKKGQKVGEFSGANKEKLEATINEL 104
```

C)

Alignment between TXNDC2 (top uc002koh.1 486aa) and TTN (bottom uc002umr.1 33423aa) score 5263

```
00038 PKSSEETIQPKEGDIPKAPEETIQSKKEDLPKS---SEKAIQPKESNIPKSSAKPIQPKL 00094
      |+ ||+ | ||+      | +   +|  ++||     ||   |+|  +| +    + |
10443 PEISEKIIPPKKPPTKVVPRKEPPAKVPEVPKKIVVEEKVRVPEEPRVPPTKVPEVLPPK 10502
```

```
00095 GNIP--KASVKPSQPKEG---DIPKAPEETIQSKKEDLPKSSEEAIQP-KEGDIPKSSA- 00147
        +|  |  | |++  |     +|+||+| +  ||  +|   +  + | |  ++||++
10503 EVVPEKKVPVPPAKKPEAPPPKVPEAPKEVVPEKKVPVPPPKKPEVPPTKVPEVPKAAVP 10562

00148 -----KPIQPKLGN------------IAKTSVKPSQPKESD-----IPKSPEETIQPKEG 00185
           + | ||  +            + +  |  + ||+ +     +|++|+| +  |+
10563 EKKVPEAIPPKPESPPPEVPEAPKEVVPEKKVPAAPPKKPEVTPVKVPEAPKEVVPEKKV 10622

00186 DIPKSSAKPIQP-KLGNIPKASVKPSQPKEG----DISKSPEEAIQPKEGDLPKSLEEAI 00240
       +|      + | |+  +|| +|   +  |       |  ||   +|+|  | +   | +
10623 PVPPPKKPEVPPTKVPEVPKVAVPEKKVPEAIPPKPESPPPEVFEEPEEVALEEPPAEVV 10682

00241 QPKEGDIPKSPEEAIQPKEGDIPKSLEEAIQPKEGD-----IPKSPEETIQPKKGD--IP 00293
      +  |   | +| +   | +  +|+    |+  |+ +     +|+ |+| +  ||    +|
10683 EEPE---PAAPPQVTVPPKKPVPEKKAPAVVAKKPELPPVKVPEVPKEVVPEKKVPLVVP 10739

00294 KSPEEAIQPKEGDIPKSPKQAIQPKEGDIPKSLEEAIPPKEI-DIPKSPEETIQPKEDDS 00352
      | ||         +|+ ||+ +  |+  +||  |  +|| ++ ++|| |    +|
10740 KKPE----APPAKVPEVPKEVVPEKKVAVPKKPE--VPPAKVPEVPKKPVLEEKPAV-PV 10792

00353 PKSLEEATPS--KEGDILKPEEETMEFPEGDKVKVILSKEDFE 00393
      |+  |   |   +| + + ||||    || +|   +  +|+ |
10793 PERAESPPPEVYEEPEEIAPEEEIA--PEEEKPVPVAEEEEPE 10833
```
