# Supplementary material for: Retrocopy contributions to the evolution of the human genome
Source: BMC Genomics. 2008 Oct 8;9:466. doi: 10.1186/1471-2164-9-466 (PMC2584115; doi:10.1186/1471-2164-9-466)
Supplement: Additional file 7 — DGCR13 and TSSK2 alignments in multiple primates. [file 1471-2164-9-466-S7.pdf]

Suppl Fig 4 – DCR13 and TSSK Protein and DNA Alignments in primates

i) DGCR13 protein alignment

```
humantra        MVFGPGGLGQPVLHPVLVLGHHQQPVLGFGSKLVVG----PVVGPQACLGVQFALSAVLA 56
chimptr         MVFGPGGLGQPVLHPVLVLGHHQQPVLGFGSKLVVG----PVVGPQACLGVQFALSVVLA 56
gorillatr       MVFGPGGLGQPVLHPVLVLGHHQQPVLGFGSKLVVG----PVVGPQACLGVQFALSAVLA 56
pongotr         MVFGPGGLGQPVLHPVLILRHHQQPVLGFGSKLVVR----LVVRPRAWLGVQFALSAILA 56
gibbontr        IVFGPGGLGQP----VLVLGHHQQPVLDFGSKLVVG----LVVGPQAWLGVQFALSTVLA 52
rhesustr        MVFGPGGLGQPVLHLVLVLGHHQQPVLGFGSKLVVGSVVGPVVGPQAWLGVQFALGAVLA 60
                :**********    **:* *******.*******      ** *:* *******..:**

humantra        LPLPLEGGRRRGFGLGGLQPRVAEDLIDVEPLADVGLQHAVDEVLALAGQVLGAREVHTV 116
chimptr         LPLPLEGGRRHGFGLGGLQPRVAEDLIDVEPLADVGLQHAVDEVLALAGQVLGAREVHTV 116
gorillatr       LPLPLEGGRRHGFGLGGLQPRVAEDLIDVEPLADVGLQHAVDEVLALAGQVLGAREVHTV 116
pongotr         LPFPLEGGPSHGFGLGGAAATSG------------------------------------- 79
gibbontr        LPLPLEGGRRHGFGLGGLQP---------------------------------------- 72
rhesustr        LPLPLEGGRRHGFGLGGLQPRVAEDLINVEPLADVRLQHAVDEVLALTGQVLGAWEVHTV 120
                **:*****  :******  .

humantra        LLLDTQHLPDVGVVIGHGAADHDVQDHAQAPDVIHLGLVGDALQHLGGCICCRPAEGLAE 176
chimptr         LLLDTQHLPDVGVVIGHGAADHDVQDHAQAPDVIHLGLVGDALQHLGGCICCRPAEGLAE 176
gorillatr       LLLDTQHLPDVGVVIGHGATDHDVQDHAQAPDVIHLGLVGDALQHLGGCICCRPAEGLAE 176
pongotr         ------------------------------------------------------------
gibbontr        ------------------------------------------------------------
rhesustr        LLLDTQHLPDVGVIVGHGAADHDVEDHTQAPDVVHLGLVGDALQHLGGCICR-------- 172


humantra        DDAPIAVPQAALGEAKVRQLDVEVLVEEKVLALEVPVDDVQVVAVLDGGGELSEHLACHV 236
chimptr         DDAPIAVPQAALGEAKVRQLDVEVLVEEKVLALEVPVDDVQVVAVLDGGGELSEHLACHV 236
gorillatr       DDVPIAVPQAALGEAKVRQLDVEVLVEEKVLALEVPVDDVQVVAVLDGGGELSEHLACHV 236
pongotr         ------------------------------------------------------------
gibbontr        ------------------------------------------------------------
rhesustr        ------------------------------------------------------------


humantra        LMQGSLALDELEEVALDAKLHDDVDPSVRGLKDLVSLDDGAVVDSCQDVHLPRKESLHKV 296
chimptr         LMQGSPALDELEEVALDAKLHDDVDPSVRGLKDLVSLDDGAVADSCQDVHLPRKESLHKV 296
gorillatr       LMQGSPALDELEEVALDAKLHDDVDPSVRGLKDLVSLDDGAVVDSCQDVHLPRKESLHKV 296
pongotr         ------------------------------------------------------------
gibbontr        ------------------------------------------------------------
rhesustr        ------------------------------------------------------------


humantra        SRCFLAVDDLDSHIELEALRVGRFDFCVGTLAKIDAYDVTLLP 339
chimptr         SRCFLAVDDLDSHIELEALRVGRFDFCVGTLAKIDAYDVTLLP 339
gorillatr       SRCFLAVDDLDSHIELEALRVGRFDFCVGTLAKIDAYDVTLLP 339
pongotr         -------------------------------------------
gibbontr        -------------------------------------------
rhesustr        -------------------------------------------
```

ii) DGCR13 – DNA alignment

```
humanrc         GGGCCATTGTCATGCTAGGTGCTTGCTTTCCCCACCTCAGCTCCGGAGATGTGATGGTCT 178
chimprc         GGGCCATTGTCATGCTAGGTGCTTGCTTTCCCCACCTCAGCTCCGGAGATGTGATGGTCT 178
gorillarc       ---CCATTGTCATGCTAGGTGCTTGCTTTCCCCACCTCAGCTCCGGAGGTGTGATGGTCT 57
pongorc         ---CCATTGTCACGCTAGGTGCTTGCTTTCCCCACCTCAGCTCCGGAGATGTGATGGTCT 57
gibbonrc        GGGCCATTGTCACGCTAGGTGCTTGCTTTCCCCACCTCAGCTCCGGAGATGTGATTGTCT 133
rhesusrc        GGGTCATCATCATGCTAGGTGCTTGCTTTCCCCACCTCAGCTCCGGAGACGTGATGGTCT 143
marmotarc       GGGCTGTTGCCATGCTAGGTGCTCGCTTTCCCCACCTCAGCTCCGGAGATGTGATGGTCT 180
                      *   ** ********** ************************  ***** ****

humanrc         TTGGCCCTGGAGGTCTCGGCCAGCCTGTCCTCCATCCTGTTCTCGTTCTCGGGCACCACC 238
chimprc         TTGGCCCTGGAGGTCTCGGCCAGCCTGTCCTCCATCCTGTTCTCGTTCTCGGGCACCACC 238
gorillarc       TTGGCCCTGGAGGTCTCGGCCAGCCTGTCCTCCATCCTGTTCTCGTTCTCGGGCACCACC 117
pongorc         TTGGCCCTGGAGGTCTCGGCCAGCCTGTCCTCCATCCTGTTCTCATTCTCAGGCACCACC 117
gibbonrc        TTGGCCCTGGAGGTCTCGGCCAGCCTGTCCTC----12nt----GTTCTCGGGCACCACC 181
rhesusrc        TTGGCCCTGGAGGTCTCGGCCAGCCTGTCCTCCACCTTGTCCTCGTTCTCGGGCACCACC 203
marmotarc       TTGGCCCTGGAAGTCTGGGCCAGCCTGTCCTCCATCCTGTCCTCGTTCTCGGGCACCACC 240
                *********** **** ***************             ** ** *********

humanrc         AGCAGCCGGTGCTGGGTTTTGGCTCCAAGCTTGTGGTCGGG----12nt----CCGGTGG 286
chimprc         AGCAGCCGGTGCTGGGTTTTGGCTCCAAGCTTGTGGTCGGG------------CCGGTGG 286
gorillarc       AGCAGCCGGTGCTGGGTTTTGGCTCCAAGCTTGTGGTCGGG------------CCGGTGG 165
pongorc         AGCAGCCGGTGCTGGGTTTTGGCTCCAAGCTTGTGGTCAGG------------CTTGTGG 165
gibbonrc        AGCAGCCGGTGCTGGATTTTGGCTCCAAGCTTGTGGTCGGG------------CTGGTGG 229
rhesusrc        AGCAGCCGGTGCTGGGTTTTGGCTCCAAGCTTGTGGTCGGGTCGGTGGTCGGGCCGGTGG 263
marmotarc       AGCGGCCGGTGCTGGTTTTTGGCTCCAAGCTTATGGTCGGG------------CTTGGGG 288
                *** *********** **************** ***** **            *  * **
```

```
humanrc         TCGGGCCTCAAGCCTGTCTTGGTGTCCAGTTTGCACTCAGCGCGGTACTTGCCCTCCCCC 346
chimprc         TCGGGCCTCAAGCCTGTCTTGGTGTCCAGTTTGCACTCAGCGTGGTACTTGCCCTCCCCC 346
gorillarc       TCGGGCCTCAAGCCTGTCTTGGTGTCCAGTTTGCACTCAGCGCGGTACTTGCCCTCCCCC 225
pongorc         TCCGGCCTCGAGCCTGGCTTGGTGTCCAGTTTGCACTCAGTGCGATACTTGCCCTCCCCT 225
gibbonrc        TCGGGCCTCAAGCCTGGCTTGGTGTCCAGTTTGCACTCAGCACGGTACTTGCCCTCCCCC 289
rhesusrc        TCGGGCCTCAAGCCTGGCTTGGTGTCCAGTTTGCACTCGGCGCGGTACTTGCCCTCCCCC 323
marmotarc       TCAGGCCTCGAGCCTGGCTTGGTGTCCAGTTTGCACTCAGCGTGGTACTTGCCCTCCCCG 348
                ** *****  ****** ********************* *   * **************

humanrc         TCCCTCTTGAAGGAGGCAGAAGACGTGGCTTTGGGCTTGGGGG-GCTGCAGCCACGAGTG 405
chimprc         TCCCTCTTGAAGGAGGCAGAAGACATGGCTTTGGGCTTGGGGG-GCTGCAGCCACGAGTG 405
gorillarc       TCCCTCTTGAAGGAGGCAGAAGACATGGCTTTGGGCTTGGGGG-GCTGCAGCCACGAGTG 284
pongorc         TTCCTCTTGAAGGAGGCCCAAGTCATGGCTTTGGGCTTGGGGGTGCTGCAGCCACGAGTG 285
gibbonrc        TCCCTCTTGAAGGAGGCAGAAGACATGGCTTTGGGCTTGGGGG-GCTGCAGCCATGAGTG 348
rhesusrc        TCCCTCTTGAAGGAGGCAGAAGACATGGCTTTGGGCTTGGGGG-GCTGCAGCCACGAGTG 382
marmotarc       TCCCTCTTGAAGGAGGCAGAAGACATGGCTTTGGGCTTGGAGG-GCTGCAGCCATGAGTG 407
                * ***************  *** * *************** ** ********** *****

humanrc         GCTGAGGATCTCATCGATGTGGAGCCGCTGGCTGACGTCGGGCTGCAGCATGCGGTAGAT 465
chimprc         GCTGAGGATCTCATCGATGTGGAGCCGCTGGCTGACGTCGGGCTGCAGCATGCGGTAGAT 465
gorillarc       GCTGAGGATCTCATCGATGTGGAGCCGCTGGCTGACGTCGGGCTGCAGCATGCGGTAGAT 344
pongorc         GCTGAGGATCTGGTCAATGTGGAGTCGCTGGCTGACATCGGGCTGCAGCATGCGGTAGAT 345
gibbonrc        GCTGAGGATCTCATCGATGTGGAGCCGCTGGCTGACGTCTGGCTGCAGCATGCGGTAGAT 408
rhesusrc        GCTGAGGATCTCATCAATGTGGAGCCGCTGGCTGACGTCAGGCTGCAGCATGCGGTAGAT 442
marmotarc       GCTGAGGATCTCGTCGATGTGGAGCCGCTGGCTGACGTCAGGCTGCAGCATGTGGTAGAT 467
                ***********  ** ******** *********** ** ************ *******

humanrc         GAGGTCCTTGCACTCGCAGGTCAGGTTCTTGGAGCGCGGGAAGTCCACACGGTGCTCCTT 525
chimprc         GAGGTCCTTGCACTCGCAGGTCAGGTTCTTGGAGCGCGGGAAGTCCACACGGTGCTCCTT 525
gorillarc       GAGGTCCTTGCACTCGCAGGTCAGGTTCTTGGAGCGCGGGAAGTCCACACGGTGCTCCTT 404
pongorc         GAGATCCTTGCACTCGCAGGTCAGGTTCTTGGAGCGCGGGAAGCCCACACGCTGCTCCTT 405
gibbonrc        GAGGTCCTTGCACTCACAGGTCAGGTTCTTGGAGCGCGGGAAGTCCACACGGTGCTCCTT 468
rhesusrc        GAGGTCCTTGCACTCACAGGTCAGGTTCTTGGAGCGTGGGAAGTCCACACGGTGCTCCTT 502
marmotarc       GAGGTCCTTGCACTCGCAGGTCAGGTTCTTGGAGCGCGGGAAGTCCACACGGTGCTCCTT 527
                *** *********** *******************  ****** ******* ********

humanrc         CTGGATACGCAGCATCTTCCTGATGTCGGAGTCGTCATAGGGCATGGAGCCGCAGACCAT 585
chimprc         CTGGATACGCAGCATCTTCCTGATGTCGGAGTCGTCATAGGGCATGGAGCCGCAGACCAT 585
gorillarc       CTGGATACGCAGCATCTTCCTGATGTCGGAGTCGTCATAGGGCATGGAGCCACAGACCAT 464
pongorc         CTGGATACGCAGCATCTTCCTGATGTTGGAGTCGTCGTAGGGCATGGAACCACAGACCAT 465
gibbonrc        CTGGATACGCAGCATCTTCCTGATGTCGGAGTCGTCGTAGGGCATGGAGCCGCAGACCAT 528
rhesusrc        CTGGATACGCAGCATCTTCCTGATGTCGGAGTCATCGTAGGGCATGGAGCCGCAGACCAT 562
marmotarc       CTGGATACGCAGCATCTTCCTGATGTCGGAGTCGTCGTAGGGCATGGAGCCGCAGACCAT 587
                ************************** ****** ** *********** ** ********

humanrc         GATGTACAGGATCACGCCCAGGCTCCAGATGTCATACACCTTGGGCTGGTAGGGGATGCT 645
chimprc         GATGTACAGGATCACGCCCAGGCTCCAGATGTCATACACCTTGGGCTGGTAGGGGATGCT 645
gorillarc       GATGTACAGGATCACGCCCAGGCTCCAGATGTCATACACCTTGGGCTGGTAGGGGATGCT 524
pongorc         GATGTAGAGGATCACACCCAGGCTCCAGATGTCATACACCTTGGGCTGGTAGGGGATGCC 525
gibbonrc        GATGTAGAGGATCACGCCCAGGCTCCAGATGTCATACACCTTGGGCTGGTAGGGGATGCC 588
rhesusrc        GATGTAGAGGATCACACCCAGGCTCCAGATGTCGTACACCTTGGGCTGGTAGGGGATGCC 622
marmotarc       GATGTAGAGGATCACGCCCAGGCTCCAGATGTCATATACCTTGGGCTGGTAAGGGATGCC 647
                ****** ******** ***************** ** ************** *******
humanrc         CTGCAGCACCTCGGGGGCTGCATATGCTGCCGACCCGCAGAAGGTCTTGCTGAGGATGAT 705
chimprc         CTGCAGCACCTCGGGGGCTGCATATGCTGCCGACCCGCAGAAGGTCTTGCTGAGGATGAT 705
gorillarc       CTGCAGCACCTCGGGGGCTGCATATGCTGCCGACCCGCAGAAGGTCTTGCTGAGGATGAT 584
pongorc         CTGCAGCACCTCGGGGGCCGCATATGCCGCCGACCCACAGAAGGTCTTGCTGAGGATGAT 585
gibbonrc        CTGCAGCACCTCGGGGGCTGCATACGCTGCCGACCCGCAGAAGGTCTTGCTGAGGATGAT 648
rhesusrc        CTGCAGCACCTCGGGGGCTGCATATGCCGCTGACCCGCAGAAGGTCTTGCTGAGGATGAT 682
marmotarc       CTGCAGCACCTCGGGGGCTGCATACGCCGCCGACCCACAGAAGGTCTTGCTGAGGATGAT 707
                ****************** ***** ** ** ***** ***********************

humanrc         GCGCCCATTGCTGTCCCGCAGGCAGCGCTTGGAGAAGCCAAAGTCAGACAGCTTGATGTT 765
chimprc         GCGCCCATTGCTGTCCCGCAGGCAGCGCTTGGAGAAGCCAAAGTCAGACAGCTTGATGTT 765
gorillarc       GTGCCCATTGCTGTCCCGCAGGCAGCGCTTGGAGAAGCCAAAGTCAGACAGCTTGATGTT 644
pongorc         GCGTCCATTGCCGTCCCGCAGGCATCGCTTGGAGAAGCCAAAGTCAGACAGCTTGATGTT 645
gibbonrc        GCGCCCATTGCTGTCCCGCAGGCAGCGCTTGGAGAAGCCAAAGTCAGACAGCTTGATGTT 708
rhesusrc        GCGCCCATTGCTGTCCCGCAGGCAGCGCTTGGAGAAGCCGAAGTCAGACAGCTTGATGTT 742
marmotarc       GCGTCCATTGCCGTCCCTCAGGCATCGCTTGGAGAAGCCAAAGTCAGACAGCTTGATGTT 767
                * * ******* ***** ****** ************** ********************

humanrc         GAAGTCCTTGTCGAGGAGAAGGTTCTCGCACTTGAGGTCCCGGTGGACGATGTCCAGGTC 825
chimprc         GAAGTCCTTGTCGAGGAGAAGGTTCTCGCACTTGAGGTCCCGGTGGACGATGTCCAGGTC 825
gorillarc       GAAGTCCTTGTCGAGGAGAAGGTTCTCGCACTTGAGGTCCCGGTGGACGATGTCCAGGTC 704
pongorc         GAAGTCTTTGTCGAGGAGAAGATTCTCGCACTTGAGGTCCCGGTGGACGATGTCCAGGTC 705
gibbonrc        GAAGTCCTTGTCGAGGAGAAGGTTCTCGCACTTGAGGTCCCGGTGGACGACGTCCAGGTC 768
rhesusrc        GAAGTCCTTGTCGAGGAGAAGGTTCTCGCACTTGAGGTCCCGGTGGACAACGTCCAGGTC 802
marmotarc       GAAGTCCTTGTCGAGGAGAAGGTTCTCGCACTTGAGGTCCCGGTGGACGACATCCAGGTC 827
                ****** ************** ************************** *  ********
```

```
humanrc         GTGGCAGTACTTGACGGCGGAGGAGAGCTGTCGGAACATCTTGCGTGCCACGTCCTCATG 885
chimprc         GTGGCAGTACTTGACGGCGGAGGAGAGCTGTCGGAACATCTTGCGTGCCACGTCCTCATG 885
gorillarc       GTGGCAGTACTTGACGGCGGAGGAGAGCTGTCGGAACATCTTGCGTGCCACGTCCTCATG 764
pongorc         GTGGCAGTACTTGACGGCCGAGGAGAGCTGTCGGAACATCTTGCGTGCCACATCCTCGGG 765
gibbonrc        GTGGCAGTACTTGACGGCCAAGGAGAGCTGTCGGAACATCTTGCGTGCCACGTCCTCGTG 828
rhesusrc        GTGGCAGTACTTGACGGCCAAGGAGAGCTGTCGGAACATCTTGCGCGCCACGTCCTCGTG 862
marmotarc       GTGGCAGTACTTGACGGCCGAGGAGAGCTGTCGGAACATCTTGCGCGCCACATCCTCCTG 887
                ******************  ************************* ***** *****  *

humanrc         CAGGGCTCCCTGGCACTTGATGAACTCGAGGAGGTCGCCCTGGACGCCAAGCTCCATGAT 945
chimprc         CAGGGCTCCCCGGCACTTGATGAACTCGAGGAGGTCGCCCTGGACGCCAAGCTCCATGAT 945
gorillarc       CAGGGCTCCCCGGCACTTGATGAACTCGAGGAGGTCGCCCTGGACGCCAAGCTCCATGAT 824
pongorc         CAGGGCCCCCCGGCACTTGATGAACTCAAGGAGGTCGCCCTGGACGCCGAGCTCCATGAT 825
gibbonrc        CAGGGCTCCCCGGCACTTGATGAACTCGAGGAGGTCGCCCTGGACGCCAAGCTCCATGAT 888
rhesusrc        CAGGGCTCCCCGGCACTTGATGAACTCGAGGAGGTCGCCCTGGACGCCAAGCTCCATGAT 922
marmotarc       CAGGGCCCCCCGGCACTTGATGAATTCGAGGAGGTCGCCCTGGACGCCAAGCTCCATGAT 947
                ****** *** ************* ** ******************** ***********

humanrc         GATGTAGATCCGTCCGTCAGAGGTCTCAAAGATCTCGTAAGTCTTGATGA-TGGAGCCGT 1004
chimprc         GATGTAGATCCGTCCGTCAGAGGTCTCAAAGATCTCGTAAGTCTTGACGA-TGGAGCCGT 1004
gorillarc       GATGTAGATCCGTCCGTCAGAGGTCTCAAAGATCTCGTAAGTCTTGATGA-TGGAGCCGT 883
pongorc         GATGTAGATCCGCCCATCAGAGGTCTCGAAGATCTCGTAGGTCTTGATGA-TGGAGCAGT 884
gibbonrc        GATGTAGATCCGCCCGTCAGAGGTCTCAAAGATCTCGTAAGTCTTGATGA-TGGAGCCGT 947
rhesusrc        GATGTAGATGCGCCCGTCAGAGGTCTCAAAGATCTCGTAGGTCTTGATGA-TGGAGCCGT 981
marmotarc       GATGTAGATCCGCCCATCAGAGGTCTCGAAGATCTCGTAGGTCTTGATGAATGGAGCAGT 1007
                ********* ** ** *********** *********** ******* ** ****** **

humanrc         GGTTGACAGTTGCCAGGATGTCCATCTCCCGAGGAAGGAATCTCTCCACAAAGTCAGTAG 1064
chimprc         GGCTGACAGTTGCCAGGATGTCCATCTCCCGAGGAAGGAATCTCTCCACAAAGTCAGTAG 1064
gorillarc       GGTTGACAGTTGCCAGGATGTCCATCTCCCGAGGAAGGAATCTCTCCACAAAGTCAGTAG 943
pongorc         GGTTGATAGTGGCCAGGATGTCTATCTCCCAAGGAAGGAACTTCTCCACAAAGTCGGTGG 944
gibbonrc        GGTTGACAGTTGCCAGGATGTCCATCTCCCGAGGAAGGAATCTCTCCACAAAGTCAGTAG 1007
rhesusrc        GGTTGACAGTTGCCAGGATGTCCATCTCCCGAGGAAGGAATCTCTCCACAAAGTCAGTGG 1041
marmotarc       GGTTGACAGTTGCCAGGATGTCCATCTCCCGAGGAAGGAACCTTTCCACAAAGTCGGTGG 1067
                ** *** *** *********** ******* *********  * *********** ** *

humanrc         GTGTTTTCTTGCGGTCGATGATCTTGACAGCCACATTGAACTTGAGGCGCTCAGAGTAGG 1124
chimprc         GTGTTTTCTTGCGGTCGATGATCTTGACAGCCACATTGAACTTGAGGCGCTCAGAGTAGG 1124
gorillarc       GTGTTTTCTTGCGGTCGATGATCTTGACAGCCACATTGAACTTGAGGCGCTCAGAGTAGG 1003
pongorc         GCATTTTCTTGCGGTCCATGATCTTGACAGCCACATTGAACTTGAGGCGCTCAGAGTAGG 1004
gibbonrc        GTGTTTTCTTGCGGTCGATGATCTTGACAGCCACATTGAACTTGAGGCGCTCAGAGTAGG 1067
rhesusrc        GTGTTTTTTTGCGGTCGATGATCTTGACAGCCACATTGAACTTGAGGCGCTCAGAGTAGG 1101
marmotarc       GCGTTTTCTTGCGGTCAATGATCTTGACAGCCACATTGAACTTGAGGCGCTCAGAGTAGG 1127
                *  **** ******** *******************************************

humanrc         CAGATTTGACTTTTGCGTAGGAACCCTTGCCAAGATTGATGCCTACGATGTAACCCTTCT 1184
chimprc         CAGATTTGACTTTTGCGTAGGAACCCTTGCCAAGATTGATGCCTACGATGTAACCCTTCT 1184
gorillarc       CAGATTTGACTTTTGCGTAGGAACCCTTGCCAAGATTGATGCCTACGATGTAACCCTTCT 1063
pongorc         CAGATTTGACTTTTGCGTAGGAACCCTTGCCAAGATTGATGCCCATGACGTAACCCTTCT 1064
gibbonrc        CAGATTTGACTTTTGCGTAGGAACCCTTGCCAAGATTGATGCCTACGATGTAACCCTTCT 1127
rhesusrc        CAGATTTGACTTTTGCGTAGGAACCCTTGCCAAGATTGATGCCTACGATGTAACCCTTCT 1161
marmotarc       CAGATTTGACTTTTGCGTAGGAACCCTTGCCGAGATTGATGCCCATGATGTAACCCTTCT 1187
                ******************************* *********** * ** ***********

humanrc         TCCTT-AGGACTGTGGCATCGTCCATGGTGCCAGGAGCGACTGGCGCCGCTGCCGTCTAC 1243
chimprc         TCCTT-AGGACTGTGGCATCGTCCATGGTGCCAGGAGCGACTGGCGCCGCTGCCGTCTAC 1243
gorillarc       TCCTT-AGGACTGTGGCATCGTCCATGGTGCCAGGAGCGACTGGCGCTGCTGCCGTCTAC 1122
pongorc         TCTTT-AGGACTGTGGCGTCGTCCATGGTGCCCGGAGCGGCTGGCGCCACTGCCGTCTAC 1123
gibbonrc        TCCTT-AGGACTGTGGCATCGTCCATGGTGCCCGGAGCGACTGGCGCCGCTGCCGTCTAC 1186
rhesusrc        TCCTT-AGGACTGTGGCATCGTCCATGG-------------------------------- 1188
marmotarc       TTCTTTAGGACCGTGGCATCGTCCATGGTGCCAGAAGCGGCTGGCGCCACTGCCGTCTAC 1247
                *  ** ***** ***** **********
```

iii) TSSK2 protein alignment

```
humantrans      MDDATVLRKKGYIVGINLGKGSYAKVKSAYSERLKFNVAVKIIDRKKTPTDFVERFLPRE 60
chimptrans      MDDATVLRKKGYIVGINLGKGSYAKVKSAYSERLKFNVAVKIIDRKKTPTDFVERFLPRE 60
gorillatra      MDDATVLRKKGYIVGINLGKGSYAKVKSAYSERLKFNVAVKIIDRKKTPTDFVERFLPRE 60
pongotra        MDDATVLKKKGYVMGINLGKGSYAKVKSAYSERLKFNVAVKIMDRKKMPTDFVEKFLPWE 60
gibbontra       MDDATVLRKKGYIVGINLGKGSYAKVKSAYSERLKFNVAVKIIDRKKTPTDFVERFLPRE 60
rhesustr        MDDATVLRKKGYIVGINLGKGSYAKVKSAYSERLKFNVAVKIIDRKKTPTDFVERFLPRE 60
                *******:****::****************************:**** ******:*** *

humantrans      MDILATVNHGSIIKTYEIFETSDGRIYIIMELGVQGDLLEFIKCQGALHEDVARKMFRQL 120
chimptrans      MDILATVSHGSIVKTYEIFETSDGRIYIIMELGVQGDLLEFIKCRGALHEDVARKMFRQL 120
gorillatra      MDILATVNHGSIIKTYEIFETSDGRIYIIMELGVQGDLLEFIKCRGALHEDVARKMFRQL 120
pongotra        IDILATINHCSIIKTYEIFETSDGRIYIIMELGVQGDLLEFIKCRGALPEDVARKMFRQL 120
gibbontra       MDILATVNHGSIIKTYEIFETSDGRIYIIMELGVQGDLLEFIKCRGALHEDVARKMFRQL 120
rhesustr        MDILATVNHGSIIKTYEIFETSDGRIYIIMELGVQGDLLEFIKCRGALHEDVARKMFRQL 120
```

```
                 :*****:.* **:*******************************:*** ***********

humantrans       SSAVKYCHDLDIVHRDLKCENLLLDKDFNIKLSDFGFSKRCLRDSNGRIILSKTFCGSAA 180
chimptrans       SSAVKYCHDLDIVHRDLKCENLLLDKDFNIKLSDFGFSKRCLRDSNGRIILSKTFCGSAA 180
gorillatra       SSAVKYCHDLDIVHRDLKCENLLLDKDFNIKLSDFGFSKRCLRDSNGHIILSKTFCGSAA 180
pongotra         SSAVKYCHDLDIVHRDLKCENLLLDKDFNIKLSDFGFSKRCLRDGNGRIILSKTFCGSAA 180
gibbontra        SLAVKYCHDLDVVHRDLKCENLLLDKDFNIKLSDFGFSKRCLRDSNGRIILSKTFCGSAA 180
rhesustr         SLAVKYCHDLDVVHRDLKCENLLLDKDFNIKLSDFGFSKRCLRDSNGRIILSKTFCGSAA 180
                 * *********:********************************.**:************

humantrans       YAAPEVLQSIPYQPKVYDIWSLGVILYIMVCGSMPYDDSDIRKMLRIQKEHRVDFPRSKN 240
chimptrans       YAAPEVLQSIPYQPKVYDIWSLGVILYIMVCGSMPYDDSDIRKMLRIQKEHRVDFPRSKN 240
gorillatra       YAAPEVLQSIPYQPKVYDIWSLGVILYIMVCGSMPYDDSDIRKMLRIQKEHRVDFPRSKN 240
pongotra         YAAPEVLQGIPYQPKVYDIWSLGVILYIMVCGSMPYDDSNIRKMLRIQKEQRVGFPRSKN 240
gibbontra        YAAPEVLQGIPYQPKVYDIWSLGVILYIMVCGSMPYDDSDIRKMLRIQKEHRVDFPRSKN 240
rhesustr         YAAPEVLQGIPYQPKVYDIWSLGVILYIMVCGSMPYDDSDIRKMLRIQKEHRVDFPRSKN 240
                 ********.******************************:**********:**.******

humantrans       LTCECKDLIYRMLQPDVSQRLHIDEILSHSWLQPPKPKATSSASFKREGEGKYRAECKLD 300
chimptrans       LTCECKDLIYRMLQPDVSQRLHIDEILSHSWLQPPKPKAMSSASFKREGEGKYHAECKLD 300
gorillatra       LTCECKDLIYRMLQPDVSQRLHIDEILSHSWLQPPKPKAMSSASFKREGEGKYRAECKLD 300
pongotra         LTCECKDLIYRMLQPDVSQRLHIDQILSHSWLQHPQAQSHDLGLLQEERGGQVSH----- 289
gibbontra        LTCECKDLIYRMLQPDVSQRLHIDEILSHSWLQPPKPKAMSSASFKREGEGKYRAECKLD 300
rhesustr         LTCECKDLIYRMLQPDVSQRLHIDEILSHSWLQPPKPKAMSSASFKREGEGKYRAECKLD 300
                 ************************:******** *:.:: . . ::.*

humantrans       TKTGLR----PDHRPDHKLGAKTQHRLLVVPENENRMEDRLAETSRAKDHHISGAEVGKA 356
chimptrans       TKTGLR----PDHRPDHKLGAKTQHRLLVVPENENRMEDRLAETSRAKDHHISGAEVGKA 356
gorillatra       TKTGLR----PDHRPDHKLGAKTQHRLLVVPENENRMEDRLAETSRAKDHHTSGAEVGKA 356
pongotra         ------------------------------------------------------------ 295
gibbontra        TKPGLR----PDHQPDHKLGAKIQHRLLVVPENED----RLAETSRAKDNHISGAEVGKA 352
rhesustr         TKPGLRPDHRPDHRPDHKLGAKTQHRLLVVPENEDKVEDRLAETSRAKDHHVSGAEVGKA 360
                                    *.: .*

humantrans       ST 358
chimptrans       ST 358
gorillatra       ST 358
pongotra         --
gibbontra        ST 354
rhesustr         ST 362
```

iv) TSSK2 – DNA alignment

```
human            ATGTAGACGGCAGCGGCGCCAGTCGCTCCTGGCACCATGGACGATGCCACAGTCCTAAGG 205
chimp            ATGTAGACGGCAGCGGCGCCAGTCGCTCCTGGCACCATGGACGATGCCACAGTCCTAAGG 205
gorilla          ATGTAGACGGCAGCAGCGCCAGTCGCTCCTGGCACCATGGACGATGCCACAGTCCTAAGG 62
pongo            ATGTAGACGGCAGTGGCGCCAGCCGCTCCGGGCACCATGGACGACGCCACAGTCCTAAAG 201
gibbon           ATGTAGACGGCAGCGGCGCCAGTCGCTCCGGGCACCATGGACGATGCCACAGTCCTAAGG 153
rhesus           ----------------------------------CCATGGACGATGCCACAGTCCTAAGG 26
marmota          ATGTAGACGGCAGTGGCGCCAGCCGCTTCTGGCACCATGGACGATGCCACGGTCCTAAAG 300
                                                  ********** ***** ******* *

human            AA-GAAGGGTTACATCGTAGGCATCAATCTTGGCAAGGGTTCCTACGCAAAAGTCAAATC 264
chimp            AA-GAAGGGTTACATCGTAGGCATCAATCTTGGCAAGGGTTCCTACGCAAAAGTCAAATC 264
gorilla          AA-GAAGGGTTACATCGTAGGCATCAATCTTGGCAAGGGTTCCTACGCAAAAGTCAAATC 121
pongo            AA-GAAGGGTTACGTCATGGGCATCAATCTTGGCAAGGGTTCCTACGCAAAAGTCAAATC 260
gibbon           AA-GAAGGGTTACATCGTAGGCATCAATCTTGGCAAGGGTTCCTACGCAAAAGTCAAATC 212
rhesus           AA-GAAGGGTTACATCGTAGGCATCAATCTTGGCAAGGGTTCCTACGCAAAAGTCAAATC 85
marmota          AAAGAAGGGTTACATCATGGGCATCAATCTCGGCAAGGGTTCCTACGCAAAAGTCAAATC 360
                 ** ********** ** * *********** *****************************

human            TGCCTACTCTGAGCGCCTCAAGTTCAATGTGGCTGTCAAGATCATCGACCGCAAGAAAAC 324
chimp            TGCCTACTCTGAGCGCCTCAAGTTCAATGTGGCTGTCAAGATCATCGACCGCAAGAAAAC 324
gorilla          TGCCTACTCTGAGCGCCTCAAGTTCAATGTGGCTGTCAAGATCATCGACCGCAAGAAAAC 181
pongo            TGCCTACTCTGAGCGCCTCAAGTTCAATGTGGCTGTCAAGATCATGGACCGCAAGAAAAT 320
gibbon           TGCCTACTCTGAGCGCCTCAAGTTCAATGTGGCTGTCAAGATCATCGACCGCAAGAAAAC 272
rhesus           TGCCTACTCTGAGCGCCTCAAGTTCAATGTGGCTGTCAAGATCATCGACCGCAAAAAAAC 145
marmota          TGCCTACTCTGAGCGCCTCAAGTTCAATGTGGCTGTCAAGATCATTGACCGCAAGAAAAC 420
                 ********************************************* ******** ****

human            ACCTACTGACTTTGTGGAGAGATTCCTTCCTCGGGAGATGGACATCCTGGCAACTGTCAA 384
chimp            ACCTACTGACTTTGTGGAGAGATTCCTTCCTCGGGAGATGGACATCCTGGCAACTGTCAG 384
gorilla          ACCTACTGACTTTGTGGAGAGATTCCTTCCTCGGGAGATGGACATCCTGGCAACTGTCAA 241
pongo            GCCCACCGACTTTGTGGAGAAGTTCCTTCCTTGGGAGATAGACATCCTGGCCACTATCAA 380
gibbon           ACCTACTGACTTTGTGGAGAGATTCCTTCCTCGGGAGATGGACATCCTGGCAACTGTCAA 332
rhesus           ACCCACTGACTTTGTGGAGAGATTCCTTCCTCGGGAGATGGACATCCTGGCAACTGTCAA 205
marmota          GCCCACCGACTTTGTGGAAAGGTTCCTTCCTCGGGAGATGGACATCCTGGCAACTGTCAA 480
                  ** ** *********** *  ********* ******* *********** *** ***

human            CCACGGCTCCAT-CATCAAGACTTACGAGATCTTTGAGACCTCTGACGGACGGATCTACA 443
```

```
chimp      CCACGGCTCCAT-CGTCAAGACTTACGAGATCTTTGAGACCTCTGACGGACGGATCTACA 443
gorilla    CCACGGCTCCAT-CATCAAGACTTACGAGATCTTTGAGACCTCTGACGGACGGATCTACA 300
pongo      CCACTGCTCCAT-CATCAAGACCTACGAGATCTTCGAGACCTCTGATGGGCGGATCTACA 439
gibbon     CCACGGCTCCAT-CATCAAGACTTACGAGATCTTTGAGACCTCTGACGGGCGGATCTACA 391
rhesus     CCACGGCTCCAT-CATCAAGACCTACGAGATCTTTGAGACCTCTGACGGGCGCATCTACA 264
marmota    CCACTGCTCCATTCATCAAGACCTACGAGATCTTCGAGACCTCTGATGGGCGGATCTACA 540
           **** ******* * ******* *********** ********** ** ** *******

human      TCATCATGGAGCTTGGCGTCCAGGGCGACCTCCTCGAGTTCATCAAGTGCCAGGGAGCCC 503
chimp      TCATCATGGAGCTTGGCGTCCAGGGCGACCTCCTCGAGTTCATCAAGTGCCGGGGAGCCC 503
gorilla    TCATCATGGAGCTTGGCGTCCAGGGCGACCTCCTCGAGTTCATCAAGTGCCGGGGAGCCC 360
pongo      TCATCATGGAGCTCGGCGTCCAGGGCGACCTCCTTGAGTTCATCAAGTGCCGGGGGGCCC 499
gibbon     TCATCATGGAGCTTGGCGTCCAGGGCGACCTCCTCGAGTTCATCAAGTGCCGGGGAGCCC 451
rhesus     TCATCATGGAGCTTGGCGTCCAGGGCGACCTCCTCGAGTTCATCAAGTGCCGGGGAGCCC 324
marmota    TCATCATGGAGCTTGGCGTCCAGGGCGACCTCCTCGAATTCATCAAGTGCCGGGGGGCCC 600
           ************* ******************* ** ************* *** ****

human      TGCATGAGGACGTGGCACGCAAGATGTTCCGACAGCTCTCCTCCGCCGTCAAGTACTGCC 563
chimp      TGCATGAGGACGTGGCACGCAAGATGTTCCGACAGCTCTCCTCCGCCGTCAAGTACTGCC 563
gorilla    TGCATGAGGACGTGGCACGCAAGATGTTCCGACAGCTCTCCTCCGCCGTCAAGTACTGCC 420
pongo      TGCCCGAGGATGTGGCACGCAAGATGTTCCGACAGCTCTCCTCGGCCGTCAAGTACTGCC 559
gibbon     TGCACGAGGACGTGGCACGCAAGATGTTCCGACAGCTCTCCTTGGCCGTCAAGTACTGCC 511
rhesus     TGCACGAGGACGTGGCGCGCAAGATGTTCCGACAGCTCTCCTTGGCCGTCAAGTACTGCC 384
marmota    TGCAGGAGGATGTGGCGCGCAAGATGTTCCGACAGCTCTCCTCGGCCGTCAAGTACTGCC 660
           ***  ***** ***** ************************  ****************

human      ACGACCTGGACATCGTCCACCGGGACCTCAAGTGCGAGAACCTTCTCCTCGACAAGGACT 623
chimp      ACGACCTGGACATCGTCCACCGGGACCTCAAGTGCGAGAACCTTCTCCTCGACAAGGACT 623
gorilla    ACGACCTGGACATCGTCCACCGGGACCTCAAGTGCGAGAACCTTCTCCTCGACAAGGACT 480
pongo      ACGACCTGGACATCGTCCACCGGGACCTCAAGTGCGAGAATCTTCTCCTCGACAAAGACT 619
gibbon     ACGACCTGGACGTCGTCCACCGGGACCTCAAGTGCGAGAACCTTCTCCTCGACAAGGACT 571
rhesus     ACGACCTGGACGTTGTCCACCGGGACCTCAAGTGCGAGAACCTTCTCCTCGACAAGGACT 444
marmota    ACGACCTGGATGTCGTCCACCGGGACCTCAAGTGCGAGAACCTTCTCCTCGACAAGGACT 720
           **********  * ************************* ************** ****

human      TCAACATCAAGCTGTCTGACTTTGGCTTCTCCAAGCGCTGCCTGCGGGACAGCAATGGGC 683
chimp      TCAACATCAAGCTGTCTGACTTTGGCTTCTCCAAGCGCTGCCTGCGGGACAGCAATGGGC 683
gorilla    TCAACATCAAGCTGTCTGACTTTGGCTTCTCCAAGCGCTGCCTGCGGGACAGCAATGGGC 540
pongo      TCAACATCAAGCTGTCTGACTTTGGCTTCTCCAAGCGATGCCTGCGGGACGGCAATGGAC 679
gibbon     TCAACATCAAGCTGTCTGACTTTGGCTTCTCCAAGCGCTGCCTGCGGGACAGCAATGGGC 631
rhesus     TCAACATCAAGCTGTCTGACTTCGGCTTCTCCAAGCGCTGCCTGCGGGACAGCAATGGGC 504
marmota    TCAACATCAAGCTGTCTGACTTTGGCTTCTCCAAGCGATGCCTGAGGGACGGCAATGGAC 780
           ********************** ************** ****** ***** ******* *
human      GCATCATCCTCAGCAAGACCTTCTGCGGGTCGGCAGCATATGCAGCCCCCGAGGTGCTGC 743
chimp      GCATCATCCTCAGCAAGACCTTCTGCGGGTCGGCAGCATATGCAGCCCCCGAGGTGCTGC 743
gorilla    ACATCATCCTCAGCAAGACCTTCTGCGGGTCGGCAGCATATGCAGCCCCCGAGGTGCTGC 600
pongo      GCATCATCCTCAGCAAGACCTTCTGTGGGTCGGCGGCATATGCGGCCCCCGAGGTGCTGC 739
gibbon     GCATCATCCTCAGCAAGACCTTCTGCGGGTCGGCAGCGTATGCAGCCCCCGAGGTGCTGC 691
rhesus     GCATCATCCTCAGCAAGACCTTCTGCGGGTCAGCGGCATATGCAGCCCCCGAGGTGCTGC 564
marmota    GCATCATCCTCAGCAAGACCTTCTGTGGGTCGGCGGCGTATGCAGCCCCCGAGGTGCTGC 840
            ************************ ***** ** ** ***** ****************

human      AGAGCATCCCCTACCAGCCCAAGGTGTATGACATCTGGAGCCTGGGCGTGATCCTGTACA 803
chimp      AGAGCATCCCCTACCAGCCCAAGGTGTATGACATCTGGAGCCTGGGCGTGATCCTGTACA 803
gorilla    AGAGCATCCCCTACCAGCCCAAGGTGTATGACATCTGGAGCCTGGGCGTGATCCTGTACA 660
pongo      AGGGCATCCCCTACCAGCCCAAGGTGTATGACATCTGGAGCCTGGGTGTGATCCTCTACA 799
gibbon     AGGGCATCCCCTACCAGCCCAAGGTGTATGACATCTGGAGCCTGGGCGTGATCCTCTACA 751
rhesus     AGGGCATCCCCTACCAGCCCAAGGTGTACGACATCTGGAGCCTGGGTGTGATCCTCTACA 624
marmota    AGGGCATCCCTTACCAGCCCAAGGTATATGACATCTGGAGCCTGGGCGTGATCCTCTACA 900
           ** ******* ************** ** ***************** ******** ****

human      TCATGGTCTGCGGCTCCATGCCCTATGACGACTCCGACATCAGGAAGATGCTGCGTATCC 863
chimp      TCATGGTCTGCGGCTCCATGCCCTATGACGACTCCGACATCAGGAAGATGCTGCGTATCC 863
gorilla    TCATGGTCTGTGGCTCCATGCCCTATGACGACTCCGACATCAGGAAGATGCTGCGTATCC 720
pongo      TCATGGTCTGTGGTTCCATGCCCTACGACGACTCCAACATCAGGAAGATGCTGCGTATCC 859
gibbon     TCATGGTCTGCGGCTCCATGCCCTACGACGACTCCGACATCAGGAAGATGCTGCGTATCC 811
rhesus     TCATGGTCTGCGGCTCCATGCCCTACGATGACTCCGACATCAGGAAGATGCTGCGTATCC 684
marmota    TCATGGTCTGCGGCTCCATGCCCTACGACGACTCCGACATCAGGAAGATGCTGCGTATCC 960
           ********** ** *********** ** ****** ***********************

human      AGAAGGAGCACCGTGTGGACTTCCCGCGCTCCAAGAACCTGACCTGCGAGTGCAAGGACC 923
chimp      AGAAGGAGCACCGTGTGGACTTCCCGCGCTCCAAGAACCTGACCTGCGAGTGCAAGGACC 923
gorilla    AGAAGGAGCACCGTGTGGACTTCCCGCGCTCCAAGAACCTGACCTGCGAGTGCAAGGACC 780
pongo      AGAAGGAGCAGCGTGTGGGCTTCCCGcGCTCCAAGAACCTGACCTGCGAGTGCAAGGATC 919
gibbon     AGAAGGAGCACCGTGTGGACTTCCCGCGCTCCAAGAACCTGACCTGTGAGTGCAAGGACC 871
rhesus     AGAAGGAGCACCGTGTGGACTTCCCACGCTCCAAGAACCTGACCTGTGAGTGCAAGGACC 744
marmota    AGAAGGAGCACCGTGTGGACTTCCCGCGCTCCAAGAACCTGACCTGCGAGTGCAAGGACC 1020
           ********** ******* ******  ****************** *********** *

human      TCATCTACCGCATGCTGCAGCCCGACGTCAGCCAGCGGCTCCACATCGATGAGATCCTCA 983
```

```
chimp           TCATCTACCGCATGCTGCAGCCCGACGTCAGCCAGCGGCTCCACATCGATGAGATCCTCA 983
gorilla         TCATCTACCGCATGCTGCAGCCCGACGTCAGCCAGCGGCTCCACATCGATGAGATCCTCA 840
pongo           TCATCTACCGCATGCTGCAGCCCGATGTCAGCCAGCGACTCCACATTGACCAGATCCTCA 979
gibbon          TCATCTACCGCATGCTGCAGCCAGACGTCAGCCAGCGGCTCCACATCGATGAGATCCTCA 931
rhesus          TCATCTACCGCATGCTGCAGCCTGACGTCAGCCAGCGGCTCCACATTGATGAGATCCTCA 804
marmota         TCATCTACCACATGCTGCAGCCTGACGTCAGCCAGCGGCTCCACATCGACGAGATCCTCA 1080
                ********* ************ ** *********** ******** **  *********

human           GCCACTCGTGGCTGCAGC-CCCCCAAGCCCAAAGCCACGTCTTCTGCCTCCTTCAAGAGG 1042
chimp           GCCACTCGTGGCTGCAGC-CCCCCAAGCCCAAAGCCATGTCTTCTGCCTCCTTCAAGAGG 1042
gorilla         GCCACTCGTGGCTGCAGC-CCCCCAAGCCCAAAGCCATGTCTTCTGCCTCCTTCAAGAGG 899
pongo           GCCACTCGTGGCTGCAGCACCCCCAAGCCCAAAGCCATGACTTGGGCCTCCTTCAAGAGG 1039
gibbon          GCCACTCATGGCTGCAGC-CCCCCAAGCCCAAAGCCATGTCTTCTGCCTCCTTCAAGAGG 990
rhesus          GCCACTCGTGGCTGCAGC-CCCCCAAGCCCAAAGCCATGTCTTCTGCCTCCTTCAAGAGG 863
marmota         GCCACTCATGGCTGCAGC-CCTCCAAGCCCAAAGCCATGTCTTCTGCCTCCTTCAAGAGG 1139
                ******* ********** ** *************** * ***  ***************

human           GAGGGGGAGGGCAAGTACCGCGCTGAGTGCAAACTGGACACCAAGACAGGCTTGAGGCCC 1102
chimp           GAGGGGGAGGGCAAGTACCACGCTGAGTGCAAACTGGACACCAAGACAGGCTTGAGGCCC 1102
gorilla         GAGGGGGAGGGCAAGTACCGCGCTGAGTGCAAACTGGACACCAAGACAGGCTTGAGGCCC 959
pongo           AAAGGGGAGGGCAAGTATCGCACTGAGTGCAAACTGGACACCAAGCCAGGCTCgAGGCCG 1099
gibbon          GAGGGGGAGGGCAAGTACCGTGCTGAGTGCAAACTGGACACCAAGCCAGGCTTGAGGCCC 1050
rhesus          GAGGGGGAGGGCAAGTACCGCGCCGAGTGCAAACTGGACACCAAGCCAGGCTTGAGGCCC 923
marmota         GACGGGGAGGGCAAGTACCACGCTGAGTGCAAACTGGACACCAAGCCAGGCTCGAGGCCT 1199
                 * ************** *   * ********************* ******  *****

human           GACCACCGGCCCGACCAC----12bp----AAGCTTGGAGCCAAAACCCAGCACCGGCTG 1150
chimp           GACCACCGGCCCGACCAC------------AAGCTTGGAGCCAAAACCCAGCACCGGCTG 1150
gorilla         GACCACCGGCCCGACCAC------------AAGCTTGGAGCCAAAACCCAGCACCGGCTG 1007
pongo           GACCACAAGCCTGACCAC------------AAGCTTGGAGCCAAAACCCAGCACCGGCTG 1147
gibbon          GACCACCAGCCCGACCAC------------AAGCTTGGAGCCAAAATCCAGCACCGGCTG 1098
rhesus          GACCACCGGCCCGACCACCGACCCGACCACAAGCTTGGAGCCAAAACCCAGCACCGGCTG 983
marmota         GACCCCAAGCCCGACCAT------------AAGCTTGGAGCCAAAAACCAGCACCGGCCG 1247
                **** *  *** *****             **************** *********** *

human           CTGGTGGTGCCCGAGAACGAGAACAGGATGGAGGACAGGCTGGCCGAGACCTCCAGGGCC 1210
chimp           CTGGTGGTGCCCGAGAACGAGAACAGGATGGAGGACAGGCTGGCCGAGACCTCCAGGGCC 1210
gorilla         CTGGTGGTGCCCGAGAACGAGAACAGGATGGAGGACAGGCTGGCCGAGACCTCCAGGGCC 1067
pongo           CTGGTGGTGCCTGAgAATGAgAACAGGATGGAGGACAGGCTGGCCGAGACCTCCAGGGCC 1207
gibbon          CTGGTGGTGCCCGAGAACGAGGACA----12bp----GGCTGGCCGAGACCTCCAGGGCC 1146
rhesus          CTGGTGGTGCCCGAGAACGAGGACAAGGTGGAGGACAGGCTGGCCGAGACCTCCAGGGCC 1043
marmota         CTGGTGGTGCCCGAGAACGAGGACAGGATGGAGGACAGGCTGGCCCAGACTTCCAGGGCC 1307
                *********** ** ** **  ***            ******** **** *********

human           AAAGACCATCACATCTCCGGAGCTGAGGTGGGGAAAGCAAGCACCTAGCATGACAATGGC 1270
chimp           AAAGACCATCACATCTCCGGAGCTGAGGTGGGGAAAGCAAGCACCTAGCATGACAATGGC 1270
gorilla         AAAGACCATCACACCTCCGGAGCTGAGGTGGGGAAAGCAAGCACCTAGCATGACAATGG- 1126
pongo           AAAGACCATCACATCTCCGGAGCTGAGGTGGGGAAAGCAAGCACCTAGCGTGACAATGG- 1266
gibbon          AAAGACAATCACATCTCCGGAGCTGAGGTGGGGAAAGCAAGCACCTAGCGTGACAATGGC 1206
rhesus          AAAGACCATCACGTCTCCGGAGCTGAGGTGGGGAAAGCAAGCACCTAGCATGATGATGAC 1103
marmota         AAAGACCATCACATCTCCGGAGCTGAGGTGGGGAAAGCGAGCACCTAGCATGGCAACAGC 1367
                ****** *****  ************************ ********** **   *
```
